# Discovery of *BEND4* as a novel single-gene prognostic marker and therapeutic target for adverse AML

**DOI:** 10.1038/s41698-026-01534-7

**Published:** 2026-06-06

**Authors:** Archisman Banerjee, Saket V. Mishra, Calvin A. Dsouza, Sameer Salunkhe, Pratik Chandrani, Shilpee Dutt

**Affiliations:** 1 Shilpee Dutt Laboratory, Advanced Centre for Treatment, Research and Education in Cancer, Tata Memorial Centre, Navi Mumbai, India; 2 Homi Bhabha National Institute, Training School Complex, Anushakti Nagar, Mumbai, India; 3 Department of Medical Oncology, Tata Memorial Centre, Mumbai, India; 4 Centre for Computational Biology, Bioinformatics and Crosstalk laboratory, Advanced Centre for Treatment, Research and Education in Cancer, Navi Mumbai, India; 5 Shilpee Dutt Laboratory, School of Life Sciences, Jawaharlal Nehru University, New Delhi, India

**Keywords:** Biomarkers, Cancer, Computational biology and bioinformatics, Oncology

## Abstract

Acute myeloid leukemia (AML) presents significant clinical challenges due to patient heterogeneity and variable treatment responses. Cytogenetic and mutation-based biomarkers dominate current prognostic classifications. However, many patients lack these canonical markers, and most are not therapeutic targets. Gene expression-based biomarkers, despite their clinical potential, remain underexplored. Here, we used transcriptome analyses of primary AML cohorts (*n* = 1338) and identified BEN domain-containing protein 4 (*BEND4*) as significantly overexpressed in adverse cytogenetic risk. Independent validation (*n* = 350) showed *BEND4* was significantly overexpressed in relapse and refractory AML patients. High *BEND4* expression was associated with poor overall survival, increased relapse risk, and was an independent risk factor in AML. Furthermore, in prognostic prediction, *BEND4* expression outperformed mutation or gene expression-based models and a Δct threshold from RT-qPCR of <12.75 differentiated adverse risk AML patients with 91% sensitivity and 81% specificity. Functionally, BEND4 perturbation modulated chemoresistance to doxorubicin and cytarabine, apoptosis, cell cycle distribution, cellular proliferation and clonogenicity, by altering several oncogenic and inflammation-related molecular pathways. In vivo, BEND4 overexpression promoted leukemogenesis and shortened survival, whereas its suppression decreased tumor burden and improved survival. This study establishes *BEND4* as a clinically relevant single gene expression-based biomarker and a potential therapeutic target in AML.

## Introduction

Acute myeloid leukemia (AML) is an aggressive and heterogeneous hematological malignancy, characterized by abnormal proliferation of undifferentiated or poorly differentiated myeloid precursors^1,2^. Based on the cytogenetic and molecular subtypes, the prognosis varies from favorable, intermediate, to adverse^3,4^. While the risk stratification determines the disease management strategy, a few biomarkers also inform the selection of a therapeutic regimen^5–7^. However, most patients still lack any of the known biomarkers^8^. Furthermore, most of the prognostically important or targetable gene biomarkers identified are either point mutation-based, like *NPM1*^9,10^, *TP53*^11^, *DNMT3A*^12^, bZIP^InDel^ *CEBPA*^13^, etc. or comprehensive chromosomal changes like *FLT3*-ITD^14,15^, 11q23.3/*KMT2A*-rearranged^16^, t(8;16)(p11.2;p13.3)/*KAT6A*-*CREBBP* gene fusion^17^ and *RUNX1*-*RUNX1T1* gene fusion^18^, etc. Although the genomic landscape in AML is constantly updating^19,20^, there are limited studies based on gene expression-based biomarkers despite the ease with which they can be measured in clinical settings^21,22^. This is partially because of the limitation of defining deregulated gene expression-based markers in leukemia due to either small cohort size or difficulties in comparing samples from different studies, which may involve different platforms or a lack of paired normal tissue. To overcome these limitations, using a modified top scoring pair (TSP)-based algorithm, we had earlier reported a 7-gene pair-based metric to prognosticate AML patients^22^. However, to broaden the scope beyond binary identification of expression-based prognostic markers and toward the identification of therapeutic targets as well, here, we used the *Z*-score-based statistical approach and identified a gene expression-based candidate prognostic marker, BEN domain-containing protein 4 (*BEND4*). We then confirmed the *BEND4* overexpression in relapse or refractory AML patients from independent cohorts. We demonstrate that the *BEND4* expression leads to poor survival, a higher risk of relapse, and is an independent risk factor for adverse AML. Furthermore, *BEND4* can predict prognostic outcome significantly better than current mutations or gene expression-based prognostication models. We therefore believe that it could be added as an additional risk factor for poor clinical outcomes. In addition, we also demonstrated that ectopic overexpression of BEND4 in vitro confers chemoresistance to the mainstay chemotherapy regimen (anthracyclines and antimetabolites class of drugs), reduces chemotherapy-induced apoptosis, promotes proliferation and clonogenicity, while knockdown of BEND4 increases sensitivity to both anthracycline and cytarabine, increases chemotherapy-induced apoptotic cell death, suppresses cell growth, and reduces clonogenic potential. Moreover, RNA sequencing after BEND4 perturbation and ChIP sequencing in BEND4-overexpressing AML cells revealed that BEND4 might be regulating multiple pathways, such as complement, IFN-γ response, and TNF-α signaling via NF-κB, that govern leukemic progression. We further extended our findings using an AML mouse model, showing that BEND4 overexpression in vivo is more tumorigenic and leads to poor survival, while knockdown of BEND4 tends to be anti-tumorigenic and has better survival. Therefore, *BEND4* holds promise as both a prognostic indicator and a potential therapeutic target in adverse AML.

## Results

### *BEND4* is highly expressed in the adverse cytogenetic risk group of AML patients

**Fig. 1:**
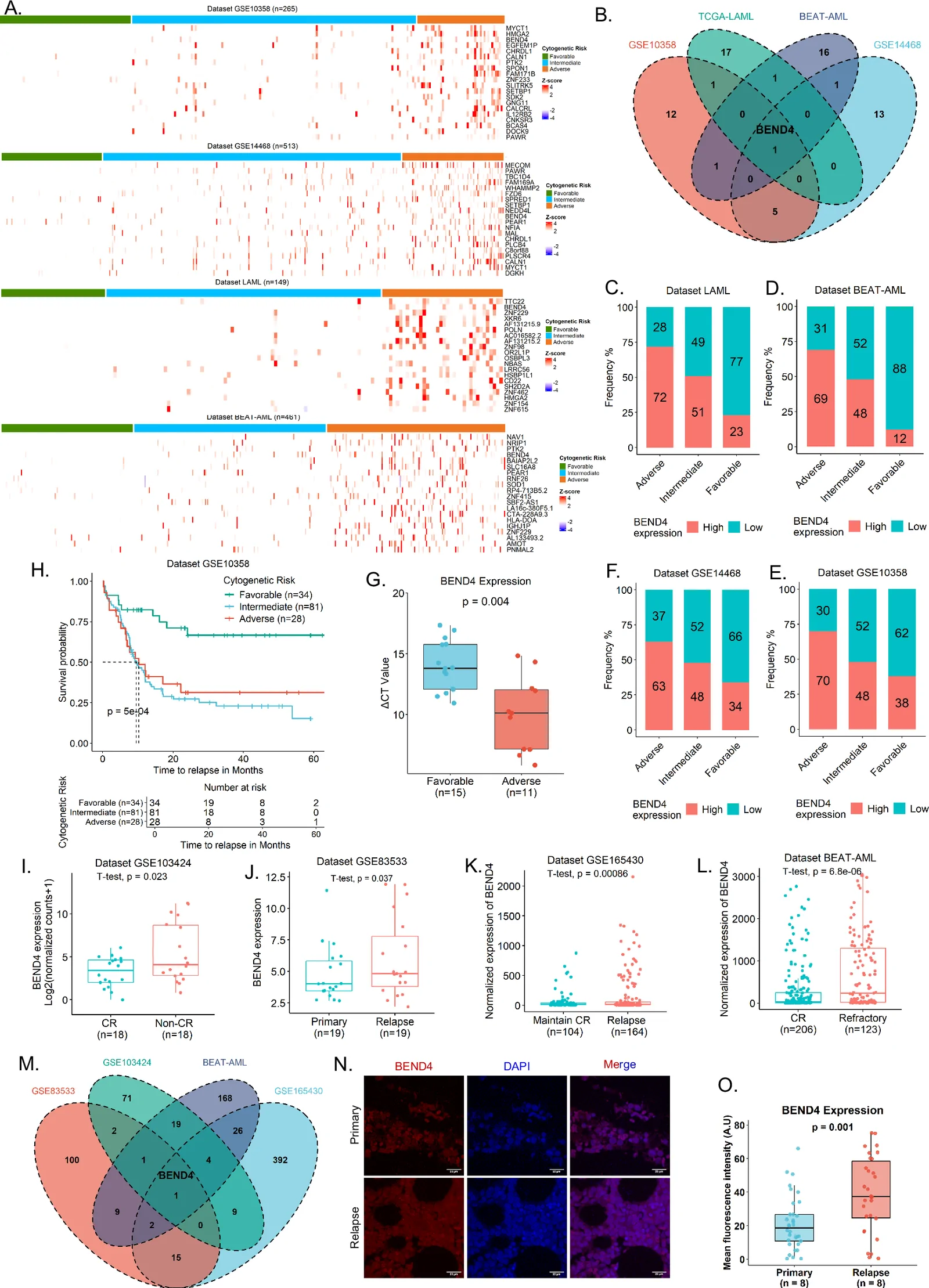
BEND4 expression correlates with cytogenetic risk and disease progression in AML across multiple datasets. **A** Heatmaps of the list of genes having *Z*-score ≥2 in the favorable (green), intermediate (blue), and adverse (orange) cytogenetic risk patients in GSE10358, GSE14468, TCGA-LAML, and BEAT-AML datasets used. The top row of each heatmap indicates the respective cytogenetic group, and the rightmost column lists the top 20 genes with a *Z*-score of ≥2 in adverse risk patients. **B**. Venn Diagram of genes represented in the heatmap of each dataset. Venn diagram showing the overlap of the top 20 recurrently upregulated genes (*Z*-score ≥ 2) in adverse-risk patients across four independent AML datasets (GSE10358, TCGA-LAML, BEAT-AML, and GSE14468). *BEND4* was the only gene consistently shared across all four cohorts, with dataset-specific genes represented in the non-overlapping regions. **C–F** Bar plot for the frequency of patients classified in each risk group according to *BEND4* expression in LAML, BEAT-AML, GSE10358, and GSE14468 datasets. Numbers in each bar represent *BEND4* high or low patient frequencies. **G**. RT-qPCR analysis of *BEND4* mRNA expression in 26 AML patients. Patients were grouped based on cytogenetic risk. Box plot of ΔCt represents the inverse of *BEND4* expression, i.e., a higher ΔCt value represents low *BEND4* gene expression. Welch’s t-test was performed to calculate the *p*-value. **H** Survival plot of event-free survival (EFS) between favorable, intermediate, and adverse prognosis groups in the mentioned dataset. Log-rank tests were performed to compare EFS, and the corresponding *p*-values were reported. Box and whiskers plot of *BEND4* expression from datasets: **I** GSE103424 (CR = 18 vs. non-CR = 18), **J** GSE83533 (Diagnosis = 19 vs. Relapse=19), **K** GSE165430 (patients who remained in CR for ≥3 years, *n* = 104 vs. patients who relapsed, *n* = 164), and **L** BEAT-AML (CR = 206 vs. Refractory = 123). Whiskers show 10 and 90 percentiles. A paired t-test for the GSE83533 dataset, and a Welch’s t-test was performed for the other datasets to calculate the *p*-values. **M** Venn diagram of up-regulated genes from the differential gene expression analysis of datasets GSE103424, GSE83533, GSE165430, BEAT-AML. **N** Representative images of immunofluorescence staining for BEND4 (Red) in paraffin- embedded bone marrow biopsies of primary (upper panel) and relapse (lower panel). DAPI (blue) was used to stain the nucleus. **O** Bar plot represents the mean fluorescence intensity of BEND4 for at least 5 images of each patient sample. Welch’s t-test was performed to obtain the *p*-value.

To identify the candidate genes whose expression is specifically associated with poor prognosis in acute myeloid leukemia (AML), we used four publicly available AML gene expression datasets—TCGA-LAML (*n* = 149), BEAT-AML (*n* = 461), GSE10358 (*n* = 265), and GSE14468 (*n* = 513). These datasets include RNA-seq (TCGA-LAML and BEAT-AML) and microarray platforms (GSE10358 and GSE14468) and comprise 1388 patients with comprehensive clinical annotations, allowing cross-cohort comparisons. The gene expression matrices were normalized using either DESeq2 for RNA-seq data or RMA normalization for microarray data. Because the datasets originate from different studies and platforms, each dataset was analyzed independently, thereby avoiding cross-study batch effects. Patients were classified into cytogenetic risk categories (adverse, intermediate, and favorable) based on the cytogenetic or ELN classification metadata accompanying each study. Considering the inherent heterogeneity among individual AML patients within these cytogenetic risk groups, we aimed to evaluate the relative expression of each gene at the individual patient level. The large sample size within each dataset enabled us to employ a *Z*-score-based statistical approach, which also mitigated cross-platform variability and addressed the absence of technical replicates or paired normal samples. It also allowed us to compare data across different distributions and was well-suited for assessing gene expression at a resolution of individual patients. Therefore, we used the normalized gene expression matrix of each dataset and transformed it to a unit standard deviation from the mean (Z-score) based matrix by calculating *Z*-scores for each gene across all patients within the same dataset. This per-gene scaling approach enabled the identification of patients with relatively elevated expression of a given gene within the cohort distribution. We defined gene expression upregulation as genes having a *Z*-score of greater than 2 units and sorted a list of genes overexpressed in adverse-risk patients across the four cohorts, prioritizing those with the highest frequency of upregulation explicitly within the adverse prognosis group (Fig. 1A). Interestingly, BEN domain-containing protein 4 (*BEND4*) was the only common gene among the top 20 upregulated genes in all four AML datasets (Fig. 1B). Sensitivity analysis using alternative *Z*-score thresholds (1.5–2.5) and permutation testing confirmed that the prioritization of *BEND4* is robust to threshold selection and unlikely to arise by chance (Fig. S1A–O). Furthermore, a high proportion of patients with adverse prognosis exhibited high *BEND4* expression (calculated from median expression) across four datasets-72% in TCGA-LAML, 69% in BEAT-AML, 70% in GSE10358, and 63% in GSE14468 (Fig. 1C–F). Furthermore, to validate our in-silico findings, we monitored the transcript level of *BEND4* in an independent cohort of 26 adult AML patients (15 favorable, and 11 adverse cytogenetic risks) accrued at Tata Memorial Hospital- Mumbai using RT- qPCR. We observed that the patients with an adverse prognosis had significantly higher expression of *BEND4* than those with a favorable prognosis (Fig. 1G). Taken together, these results identified *BEND4* as a novel gene in AML, consistently overexpressed in the adverse cytogenetic risk group of patients across multiple independent cohorts, suggestive of a strong association between its expression and adverse cytogenetic risk. This prompted us to investigate whether elevated *BEND4* expression is also associated with relapse or refractory disease.

### High *BEND4* expression is associated with relapse or refractory AML

Since patients in the adverse cytogenetic risk group are known to be more prone to either refractory disease or relapse^23^, as reflected by their poor event-free survival (Fig. 1H), we evaluated *BEND4* expression in independent AML patient cohorts with relapse or refractory AML. We found significantly higher expression of *BEND4* in three independent datasets, GSE103424 (*n* = 36)^24^, GSE83533(n = 38)^25^, GSE165430(*n* = 268)^26^, and in previously used BEAT-AML(*n* = 329) (Fig. 1I-L). Importantly, the differential gene expression analysis between complete remission (CR) and non-CR in GSE103424, primary and relapse in GSE83533, maintain CR for at least 3 years and relapse in GSE165430, CR and refractory in BEAT-AML, showed *BEND4* as the only common gene differentially upregulated (Log2Foldchange ≥ 1, *P*-value and adjusted *P*-value ≤ 0.05) in relapse or refractory AML patients (Fig. 1M). To further validate our finding, we assessed the expression of BEND4 protein in bone marrow biopsies of 8 primary and relapsed AML patients by IHC-IF (Fig. 1N). We found significantly higher (*p* = 0.001) BEND4 protein expression in relapse AML bone marrow biopsy as compared to their corresponding de novo counterpart (Fig. 1O). Together, these data confirm that *BEND4* is significantly overexpressed in refractory and relapsed AML patients.

### *BEND4* expression is a potential prognostic marker in AML

**Fig. 2:**
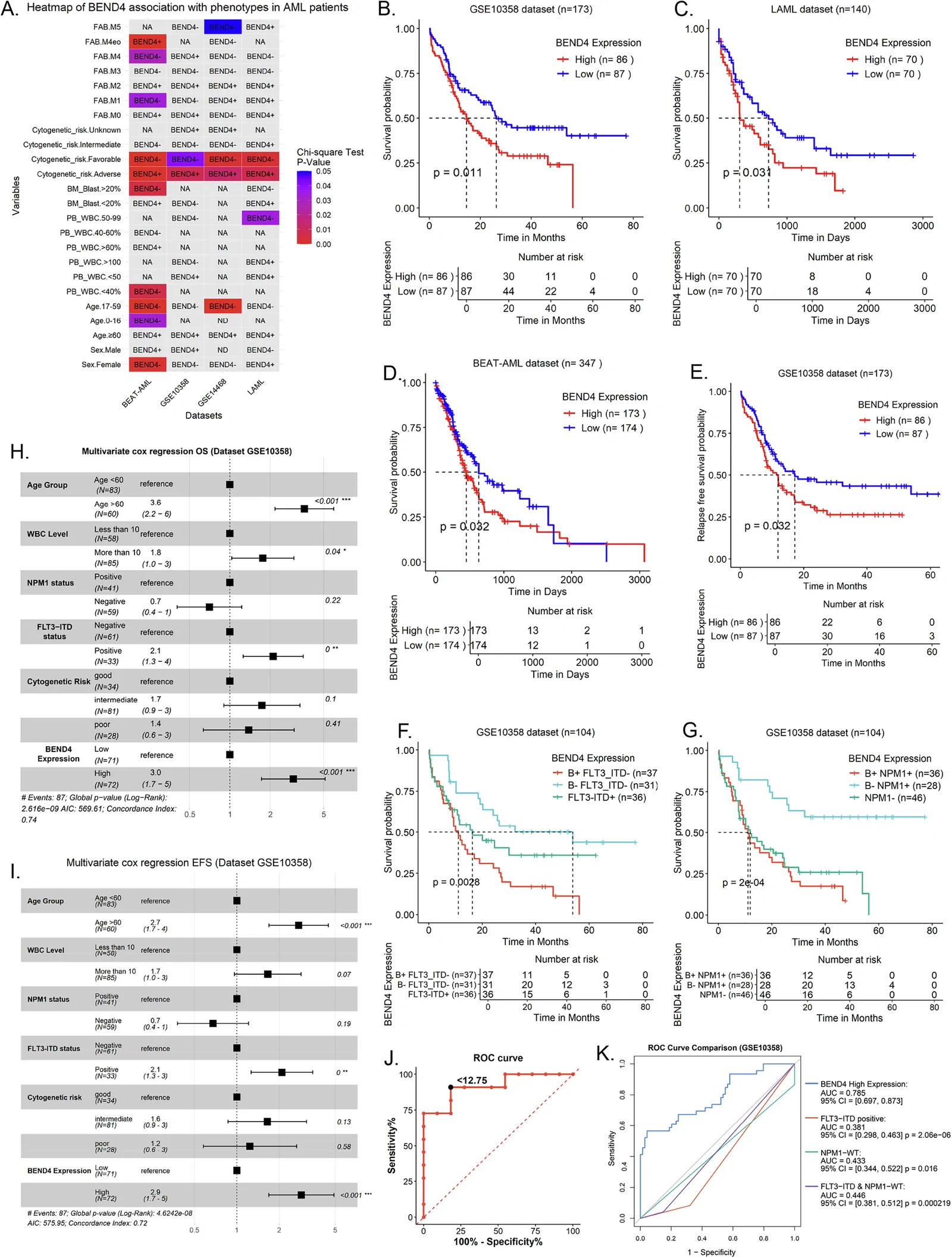
High BEND4 expression is associated with reduced survival in AML patients. **A** Bivariate analysis showing the association of *BEND4* expression levels with clinical characteristics in four different datasets (BEAT-AML, GSE10358, GSE14468, LAML). BEND4+ represents high *BEND4* expression, and BEND4- represents low *BEND4* expression. Chi-square tests were performed to obtain *p*-values. **B–D** Kaplan Meier curve for overall survival (OS) of patients from the GSE10358, TCGA- LAML, and BeatAML datasets according to *BEND4* high and low expression. Log-rank tests were performed to compare OS, and the corresponding *p*-values were reported. **E** Kaplan Meier curve for event-free or relapse free survival (EFS) of patients from the GSE10358 dataset according to *BEND4* high and low expression. Log-rank tests were performed to compare EFS, and the corresponding *p*-values were reported. **F** Kaplan Meier curve for overall survival (OS) of patients who are *FLT3*-ITD positive (*FLT3*-ITD+) or BEND4 high and low expression in *FLT3*-ITD negative (B+ *FLT3*-ITD- and B- *FLT3*-ITD-) from the GSE10358 dataset. Log-rank tests were performed to compare OS, and the corresponding *p*-values were reported. **G** Kaplan Meier curve for overall survival (OS) of patients who are *NPM1* negative (*NPM1*-) or *BEND4* high and low expression in *NPM1* positive (B+ *NPM1*+ and B- *NPM1*+) from the GSE10358 dataset. Log-rank tests were performed to compare OS, and the corresponding *p*-values were reported. **H** Forest plot for multivariate analysis of overall survival in GSE10358 dataset. Hazard ratio (HR), confidence intervals (CI), and *p*-value were calculated using Cox regression. **I** Forest plot for multivariate analysis of EFS in the GSE10358 dataset. Hazard ratio (HR), confidence intervals (CI), and *p*-value were calculated using Cox regression. **J** Receiver Operating Characteristic (ROC) curve of *BEND4* mRNA expression measured by RT-qPCR in peripheral blood blasts from 26 AML patients (15 favorable and 11 adverse cytogenetic risk). The area under the curve (AUC) was 0.917, and the optimal ΔCt threshold of <12.75 was identified as a potential prognostic cutoff for stratifying adverse from favorable cytogenetic risk, with 91% sensitivity and 81% specificity. The optimal cutoff was determined using the Youden Index. **K** ROC analysis comparing *BEND4* expression with *FLT3*-ITD and *NPM1* mutation status for predicting cytogenetic risk. In GSE10358 dataset BEND4 showed superior performance (AUC = 0.785) compared to *FLT3*-ITD (AUC = 0.381), *NPM1*-WT (AUC = 0.433), and combined *FLT3*-ITD & *NPM1*-WT (AUC = 0.446), with all comparisons statistically significant (*p* ≤ 0.05) calculated using DeLong’s test for two correlated ROC curves.

To understand the association of *BEND4* expression with clinical characteristics, we performed a bivariate association analysis of its expression with AML patients’ clinical characteristics, comprising gender, age group, WBC count, blast percentage, FAB classification, and cytogenetic risk categories in four previously used AML datasets (TCGA-LAML, BeatAML, GSE10358, and GSE14468) (Fig. 2A and Supplementary Data 1). Patients exhibiting high *BEND4* expression, calculated from the median expression, showed a significant association (*p* ≤ 0.05) with adverse cytogenetic risk group patients in all four datasets. In contrast, low *BEND4* expression was significantly associated with favorable risk group patients in those datasets. We did not find an association between other phenotypic characteristics and *BEND4* expression. This further corroborates its association with adverse prognosis. To study the survival significance, we then sought the BloodSpot 3.0 database^27^ and found that patients with high *BEND4* (gene identifier: 230896_at) expression showed significantly poor survival (Fig. S2A). Concordantly, the Kaplan–Meier survival analysis from the three AML patient gene expression datasets (GSE10358, TCGA-LAML, and BEAT-AML) showed that patients with high *BEND4* expression had poor overall survival (Log-rank *p*-value 0.011, 0.031, and 0.032, respectively, for GSE10358, TCGA-LAML, and BEAT-AML datasets) (Fig. 2B–D). Given that we observed high *BEND4* expression in relapsed AML patients, we investigated whether *BEND4* expression can predict relapse-free or event-free survival (EFS). Among all the datasets analyzed, GSE10358 was the only cohort that included EFS information. Interestingly, in this dataset, patients with high *BEND4* expression exhibited significantly shorter EFS (*p* = 0.032), further indicating the association of *BEND4* with adverse prognosis and possible chemoresistance (Fig. 2E).

Since the presence or absence of *FLT3* and *NPM1* mutations is considered as gold standard prognostic markers for AML, we wanted to analyze whether *BEND4* expression in *FLT3*-ITD negative or *NPM1* positive (which are considered favorable prognosis markers) AML patients can further predict the overall survival. For this, we first performed survival analyses of the patients with *FLT3* and *NPM1* mutations. Surprisingly, except for the *FLT3* mutation in the BEAT-AML dataset, both *FLT3* and *NPM1* mutations alone were not statistically significant in identifying overall survival over time in the GSE10358, TCGA-LAML, and BEAT-AML datasets (Fig. S3A–F). Interestingly, however, high *BEND4* expression in *FLT3*-ITD negative patients could identify statistically significantly worse overall survival (*p* = 0.0028) among respective sub-groups (Figs. 2F and S4A, B). *BEND4* high expression in *NPM1* positive patients, on the other hand, could differentiate significantly (*p* < 0.001) in the GSE10358 dataset (Fig. 2G), but did not show a survival difference in TCGA-LAML and BEAT-AML datasets (Figs. S4C, D). This lack of significance in TCGA-LAML and BEAT-AML datasets may be attributed to the limited number of patients with concurrent high *BEND4* expression and *NPM1* mutation in those cohorts (*n* = 11 and *n* = 13, respectively). Importantly, to understand whether *BEND4* expression is an independent risk factor of AML, we performed multivariable analyses, adjusted for age group, WBC counts, cytogenetic risk, *NPM1* and *FLT3*-ITD mutation status, as a time-dependent variable. The multivariate cox regression analysis confirms the independent association of high *BEND4* expression with a higher probability of worse overall survival [(Hazard ratio = 3.0, (95% CI, 1.7–5.0), *p* = <0.001)] (Fig. 2H) and relapse free survival [(Hazard ratio = 2.9, (95% CI, 1.7–5.0), *p* = <0.001)] (Fig. 2I) in the GSE10358 dataset. For an initial assessment of clinical applicability of *BEND4* mRNA expression as a potential prognostic marker for AML, we then performed ROC curve analysis using the previously shown RT-qPCR data (Fig. 1G) to measure the accuracy of *BEND4* expression-based delimitation of favorable and adverse risk among AML patients. ROC curve analysis of qPCR expression data showed a significant Area under the curve (AUC), i.e., 0.917, and a Δct value of <12.75 as a preliminary cut-off that can differentiate adverse risk patients with 81% sensitivity and 91% specificity, positive likelihood ratio (LR+) 5.0 (95% CI: 1.41–17.76), negative likelihood ratio (LR-) 0.11 (95% CI: 0.02–0.74), and bootstrap-validated AUC 0.915 (95% CI: 0.75–1.00; 1000 iterations) (Figs. 2J,S5A, B and Table S1). Considering the limited cohort size (*n* = 26), this threshold should be considered an exploratory estimate, and prospective validation in larger, independent AML cohorts will be required to establish a clinically applicable cutoff. To corroborate this, we also performed a similar ROC analysis to compare with *FLT3*-ITD and *NPM1* mutations using the previous four datasets (TCGA-LAML, BEAT-AML, GSE10358, and GSE14468). We observed that *BEND4* expression significantly distinguished between adverse and favorable cytogenetic groups with significant AUC values of 0.785 (GSE10358), 0.718 (GSE14468), 0.812 (TCGA-LAML), and 0.831 (BEAT-AML) (Figs. 2K and S6A–C). Moreover, *BEND4* consistently outperformed *FLT3* positive (*p* ≤ 0.05), *NPM1* negative (*p* ≤ 0.05), and combined *FLT3* positive and *NPM1* negative (*p* ≤ 0.05) classifiers across all the datasets (Figs. 2K and S6A–C). Furthermore, we also compared the adverse cytogenetic classification performance of *BEND4* with previously reported gene expression-based prognostic markers such as *BAALC*^28^, *ERG*^29^, *MN1*^30,31^, and *GFI1*^32^. Interestingly, we observed that *BEND4* expression identified adverse prognosis significantly (*p* ≤ 0.05) better than each of those genes across all the datasets (Fig. S7A–D). Furthermore, comparison with ELN2017 risk classification in the BEAT-AML cohort showed that *BEND4* achieved comparable predictive performance for overall survival using time-dependent ROC analysis (Fig. S8A) and showed modest improvement in risk stratification when combined with ELN, as suggested by positive NRI estimates at 2- and 3-year OS and decision-curve analyses (Fig. S8B, C). Together, these findings support a strong association of *BEND4* overexpression with adverse prognosis in AML and highlight its potential as a gene expression-based prognostic marker for AML.

### BEND4 expression alters chemoresistance in vitro

**Fig. 3:**
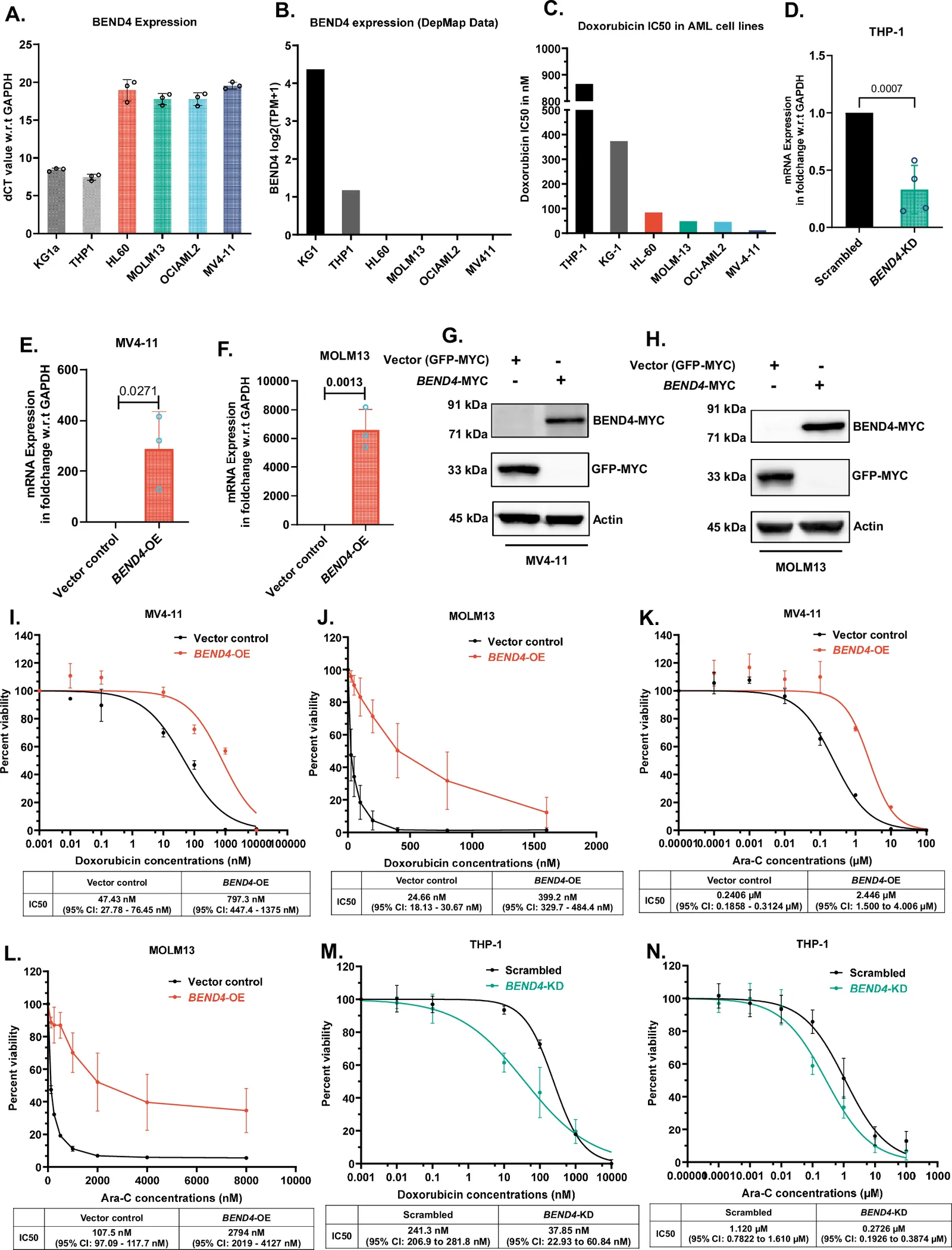
BEND4 expression modulates drug resistance in AML cell lines. **A** Bar graph of ΔCt values from RT-qPCR of *BEND4* mRNA expression in KG1a, THP-1, HL-60, MOLM13, OCI-AML2 and MV4-11 cell lines. *GAPDH* was used as an internal control. Data represent the mean ± SD of 3 independent experiments. **B** Bar plot of *BEND4* transcript levels in log2(TPM + 1) of the mentioned cell lines from the DepMap database. **C** Bar plot showing IC50 of doxorubicin in the mentioned AML cell lines from the Genomics of Drug Sensitivity in Cancer database. **D** Bar plot showing RT-qPCR for mRNA levels of *BEND4* in the THP-1 cells with stable BEND4 knockdown (BEND4-KD) compared to THP-1 cells transfected with a scrambled control. The values are in fold-change with respect to *GAPDH* expression. Data represent the mean ± SD of 4 independent experiments. The *p*-value weas calculated using t-tests. **E**, **F** Bar plot of RT-qPCR for mRNA level of *BEND4* in MV4-11 and MOLM13 cells stably transfected with BEND4 construct or vector control. The values are in fold-change with respect to *GAPDH* expression. Data represent the mean ± SD of 3 independent experiments. The *p*-value weas calculated using t-tests. Immunoblot of MV4-11 (**G**) and MOLM13 (**H**) cells, stably expressing BEND4 with MYC- Flag tag or the vector control expressing GFP with MYC-Flag tag. Western blot using an anti- MYC tag antibody is shown, identifying bands of appropriate molecular weight in each lane. Western blots are representative of at least three independent experiments. **I**, **J** Percent viability plot for the MTT assay after 48 h of doxorubicin treatment to the MV4-11 and MOLM13 BEND4 overexpressing cells with vector control. Results in the line graph are the composite data from three independent experiments (mean ± SD). IC_50_ values of each cell type along with 95% CIs were reported at the bottom of the graph. **K**, **L** Percent viability plot for the MTT assay after 48 h of cytarabine (Ara-C) treatment to the MV4-11 and MOLM13 BEND4 overexpressing cells with vector control. Results in the line graph are the composite data from three independent experiments (mean ± SD). IC_50_ values of each cell type along with 95% CIs were reported at the bottom of the graph. **M**, **N** Percent viability plot for the MTT assay after 48 h of doxorubicin and cytarabine (Ara-C) treatment to THP-1 BEND4 knockdown cells with scrambled control. Results in the line graph are the composite data from three independent experiments (mean ± SD). IC_50_ values of each cell type along with 95% CIs were reported at the bottom of the graph.

**Fig. 4:**
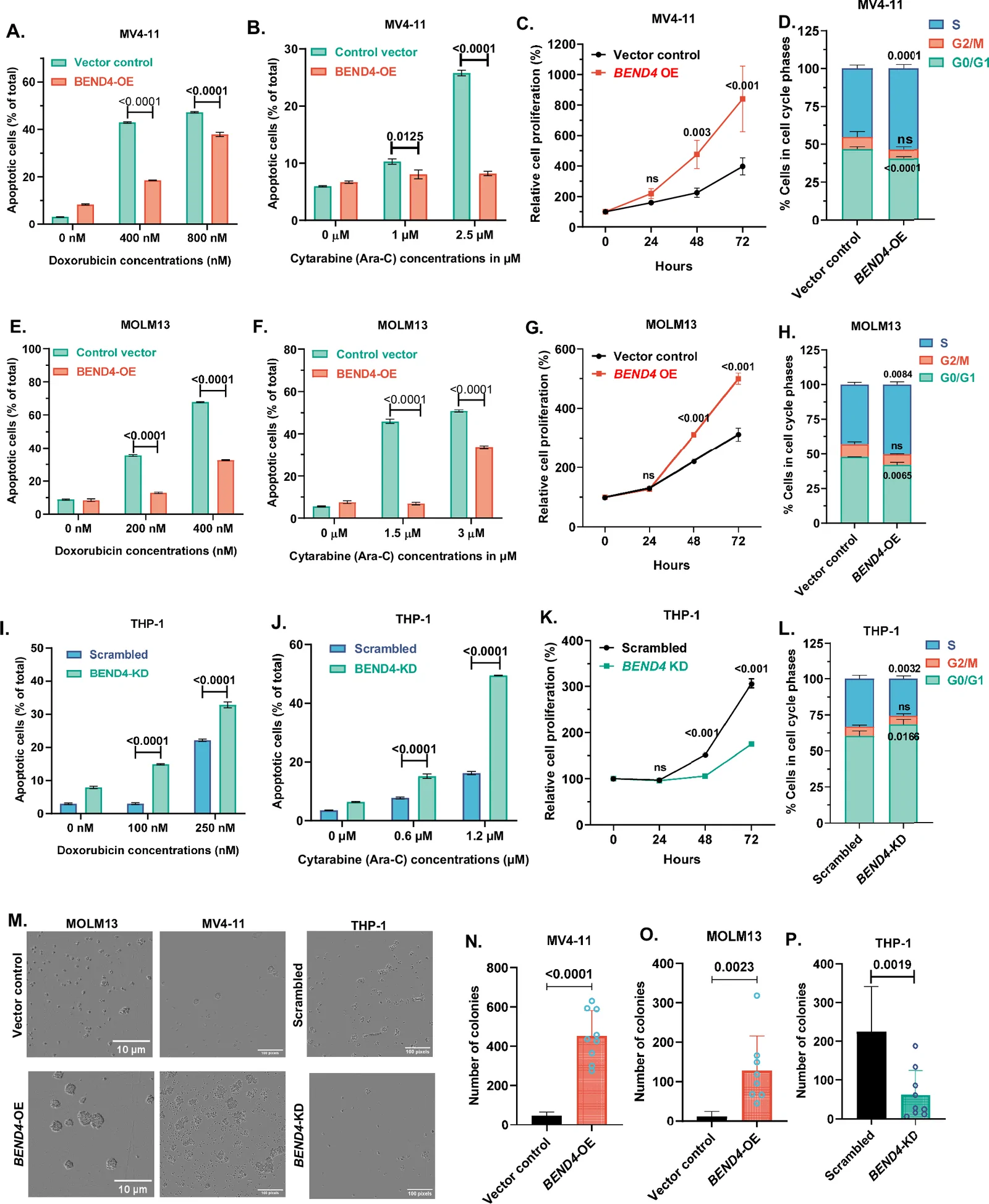
BEND4 modulates apoptosis, cell proliferation, cell cycle distribution, and clonogenic capacity in AML cell lines. **A**, **B** Annexin V/7-AAD-based apoptosis assay showing the percentage of apoptotic cells (% of total) in MV4-11 BEND4-OE and vector control cells treated with increasing concentrations of (**A**) doxorubicin (0, 400, and 800 nM) and (**B**) cytarabine (Ara-C; 0, 1, and 2.5 μM) for 48 h. Data represent the mean ± SD of three independent experiments. *p*-values were calculated using unpaired t-tests. **C** MTT-based relative cell proliferation assay showing proliferation (%) of MV4-11 BEND4-OE and vector control cells at 0, 24, 48, and 72 h. Relative cell proliferation was calculated as (Aₓh/A₀h) × 100. Data represent the mean ± SD of three independent experiments. *p*-values were calculated using multiple t-tests. **D** Cell cycle analysis by propidium iodide (PI) staining and flow cytometry showing the percentage of MV4-11 BEND4-OE and vector control cells in S, G2/M, and G0/G1 phases. Data represent the mean ± SD of three independent experiments. *p*-values were calculated using unpaired t-tests; ns = not significant. **E**, **F** Annexin V/7-AAD-based apoptosis assay showing the percentage of apoptotic cells (% of total) in MOLM13 BEND4-OE and control vector cells treated with increasing concentrations of **E** doxorubicin (0, 200, and 400 nM) and **F** cytarabine (Ara-C; 0, 1.5, and 3 μM) for 48 h. Data represent the mean ± SD of three independent experiments. *p*-values were calculated using unpaired t-tests. **G** MTT-based relative cell proliferation assay showing proliferation (%) of MOLM13 BEND4-OE and control vector cells at 0, 24, 48, and 72 h. Relative cell proliferation was calculated as (Aₓh/A₀h) × 100. Data represent the mean ± SD of three independent experiments. *p*-values were calculated using multiple t-tests. **H** Cell cycle analysis by PI staining and flow cytometry showing the percentage of MOLM13 BEND4-OE and control vector cells in S, G2/M, and G0/G1 phases. Data represent the mean ± SD of three independent experiments. *p*-values were calculated using unpaired t-tests; ns = not significant. **I**, **J** Annexin V/7-AAD-based apoptosis assay showing the percentage of apoptotic cells (% of total) in THP-1 BEND4-KD and scrambled control cells treated with increasing concentrations of **I** doxorubicin (0, 100, and 250 nM) and **J** cytarabine (Ara-C; 0, 0.6, and 1.2 μM) for 48 h. Data represent the mean ± SD of three independent experiments. *p*-values were calculated using unpaired t-tests. **K** MTT-based relative cell proliferation assay showing proliferation (%) of THP-1 BEND4-KD and scrambled control cells at 0, 24, 48, and 72 h. Relative cell proliferation was calculated as (Aₓh/A₀h) × 100. Data represent the mean ± SD of three independent experiments. *p*-values were calculated using multiple t-tests. **L** Cell cycle analysis by PI staining and flow cytometry showing the percentage of THP-1 BEND4-KD and scrambled control cells in S, G2/M, and G0/G1 phases. Data represent the mean ± SD of three independent experiments. *p*-values were calculated using unpaired t-tests; ns = not significant. **M** Representative micrographs from IncuCyte® Live-Cell Analysis System imaging at ×4 magnification showing colony morphology for the clonogenic assay of MOLM13 BEND4-OE, MV4-11 BEND4-OE, and THP-1 BEND4-KD cells and their respective controls after 12–14 days. Scale bar = 10 μm or 100 pixels. Quantification of colony number from the clonogenic assay for **N** MV4-11 BEND4-OE versus vector control, **O** MOLM13 BEND4-OE versus vector control, and **P** THP-1 BEND4-KD versus scrambled control. Data represent the mean ± SD of three biological replicates. *p*-values were calculated using unpaired t-tests. For apoptosis assays, pLenti-c-Myc-DDK-P2A-Puro was used as a vector control for MV4-11 and MOLM13 to avoid spectral overlap with FITC-conjugated Annexin V.

To understand the effect of BEND4 expression on chemotherapeutics, we first checked the transcript level of *BEND4* in 6 different AML human cell lines. Out of the 6 cell lines, KG1a and THP-1 cells showed higher *BEND4* transcript levels, whereas the rest of the 4 cell lines tested, namely, MV4-11, MOLM13, HL-60, and OCI-AML2, had low *BEND4* mRNA expression (Fig. 3A). We corroborated our findings with the transcript level of *BEND4* from the DepMap database^33^ selected for AML lineage (Fig. S12A). Importantly, DepMap data also showed the same pattern of *BEND4* expression in the 6 cell lines that we had used (Fig. 3B). To correlate with chemoresistance, we referred to the Genomics of Drug Sensitivity in Cancer database^34^ for the IC50 values of doxorubicin, an anthracycline class of chemotherapeutic, across these six AML cell lines. We found that doxorubicin IC50 was higher only in KG1a and THP-1 cells—the two cell lines that exhibited elevated *BEND4* expression (Fig. 3C). For further understanding of BEND4’s relation to chemoresistance and subsequent phenotypic changes, we chose to stably knock down BEND4 in an AML cell line with higher BEND4 expression (THP-1 BEND4-KD) and overexpress BEND4 in the AML cell lines with lower BEND4 expression (MV4-11 BEND4-OE and MOLM13 BEND4-OE). BEND4 knockdown (Fig. 3D) and overexpression (Fig. 3E, F) were confirmed at the transcript level by qPCR. Overexpression was also confirmed at the protein level using anti-MYC antibody (Fig. 3G, H), respectively. MV4-11 and MOLM13 BEND4 overexpressed cells were then treated with two of the most used classes of chemotherapeutics in AML, anthracycline (doxorubicin) and antimetabolite (cytarabine or Ara-C)^4^, for 48 h. Post-drug treatment, MTT-based cell viability assay revealed that BEND4 overexpressing MV4-11 and MOLM13 cells showed about 16-fold higher resistance to doxorubicin (Fig. 3I, J) compared to the cells having vector control. Whereas, in the case of Ara-C, resistance was about 10-fold higher in MV4-11 and 26-fold higher in MOLM13 (Fig. 3K, L), compared to their respective vector control cells. Notably, the IC_50_ values of BEND4-overexpressing cells (Table S2) shifted toward clinically achievable peak plasma concentrations (Cmax) of doxorubicin (~4–8 µM)^35^ and cytarabine steady-state concentration (Css) (~2–5 µM)^36^ or high-dose cytarabine Cmax (10–50 µM)^37^, suggesting that BEND4 expression reduces sensitivity to standard AML chemotherapy within clinically relevant exposure ranges. Similarly, to assess the effect of BEND4 knockdown in the AML cell line, we treated THP-1 BEND4-KD and THP-1 scrambled control cells with doxorubicin and Ara-C for 48 h. Cell viability assay after the drug treatment revealed that THP-1 BEND4-KD cells showed 6-fold increased sensitivity to doxorubicin (Figs. 3M), and 4-fold increase in sensitivity to Ara-C (Fig. 3N) compared to the THP-1 scrambled control cells. Consistent with these observations, Annexin V–based apoptosis assay demonstrated that upon treatment with increasing concentrations of doxorubicin, BEND4-overexpressing MV4-11 and MOLM13 cells exhibited significantly lower levels of apoptotic cells compared with vector control cells (Figs. 4A, E and S9A, C). Similarly, treatment with cytarabine (Ara-C) resulted in significantly reduced apoptosis in BEND4-overexpressing MV4-11 and MOLM13 cells compared with vector control cells in a dose-dependent manner (Figs. 4B, F and S9B, D), indicating suppression of chemotherapy-induced cell death. In contrast, BEND4 knockdown in THP-1 cells significantly increased apoptosis following treatment with increasing concentrations of both doxorubicin and Ara-C compared with scrambled control cells (Figs. 4I, J and S9E, F). Furthermore, given that anthracycline and cytarabine are commonly used in combination for AML treatment, Bliss and ZIP synergy analyses showed synergistic interactions between doxorubicin and cytarabine across tested dose combinations, with comparable synergy scores observed between control and BEND4-perturbed cells (Fig. S10A–H), indicating that BEND4 perturbation modulates intrinsic drug sensitivity without altering the synergistic interaction between the two agents. Altogether, these findings indicate that BEND4 promotes chemoresistance in AML cells by attenuating chemotherapy-induced apoptosis.

### BEND4 expression leads to altered cell proliferation and colony formation

Since we found that BEND4 overexpression leads to chemoresistance in AML cells, we further examined the other tumorigenic properties of AML cells post-BEND4 genetic perturbation. MTT-based proliferation assay showed that over-expression of BEND4 significantly increased the proliferation rate of the MV4-11 BEND4-OE (*p*-value < 0.001) and MOLM13 BEND4-OE (*p*-value < 0.001) cells (Fig. 4C, G), compared to their respective vector control cells. In contrast, knockdown of BEND4 significantly decreased (*p*-value < 0.001) the proliferation of THP-1 BEND4-KD cells compared to the scrambled control (Fig. 4K). To determine whether these changes in proliferation were associated with alterations in cell-cycle progression, we performed cell-cycle analysis by flow cytometry. BEND4-overexpressing MV4-11 and MOLM13 cells both showed significantly decreased G0/G1 populations (MV4-11: *p* < 0.0001; MOLM13: *p* = 0.0065) and increased S-phase populations (MV4-11: *p* = 0.0001; MOLM13: *p* = 0.0084) compared to vector controls, with G2/M phases remaining largely unchanged (Fig. 4D, H). Conversely, THP-1 BEND4-KD cells significantly increased the proportion of cells in the G0/G1 phase (*p* = 0.0166) and reduced the S-phase population (*p* = 0.0032) relative to scrambled controls, with no significant change in the G2/M phase (Fig. 4L). Together, these results suggest that BEND4 enhances AML cell proliferation by promoting G1–S phase transition, thereby accelerating cell-cycle progression. We further assessed the clonogenic potential of AML cells following BEND4 overexpression and knockdown. BEND4 overexpression led to a significant increase in colony formation in both MV4-11 and MOLM13 cells, as evidenced by a significant increase in the number of colonies (Fig. 4M–O), mean colony area (Fig. S11A, B), colony-forming efficiency (Fig. S11C, D), median colony area, and total integrated colony area (Fig. S11I- L) compared to vector control cells. Analysis of colony size distribution further revealed a pronounced rightward shift toward larger colonies in BEND4-OE cells, indicating enhanced clonogenic growth (Fig. S11G, H). Conversely, knockdown of BEND4 in THP-1 cells resulted in a significant reduction in the number of colonies (Fig. 4M, P), mean colony area and colony-forming efficiency (Fig. S11E, F), median colony area, and total integrated colony area (Fig. S11M, N) relative to scrambled controls, accompanied by a shift toward smaller colonies in the size distribution (Fig. S11O). Collectively, these findings demonstrate that BEND4 promotes AML cell growth by enhancing proliferation, accelerating G1–S cell-cycle progression, and increasing clonogenic capacity.

### BEND4 regulates inflammatory and oncogenic signaling pathways through direct chromatin occupancy in AML

**Fig. 5:**
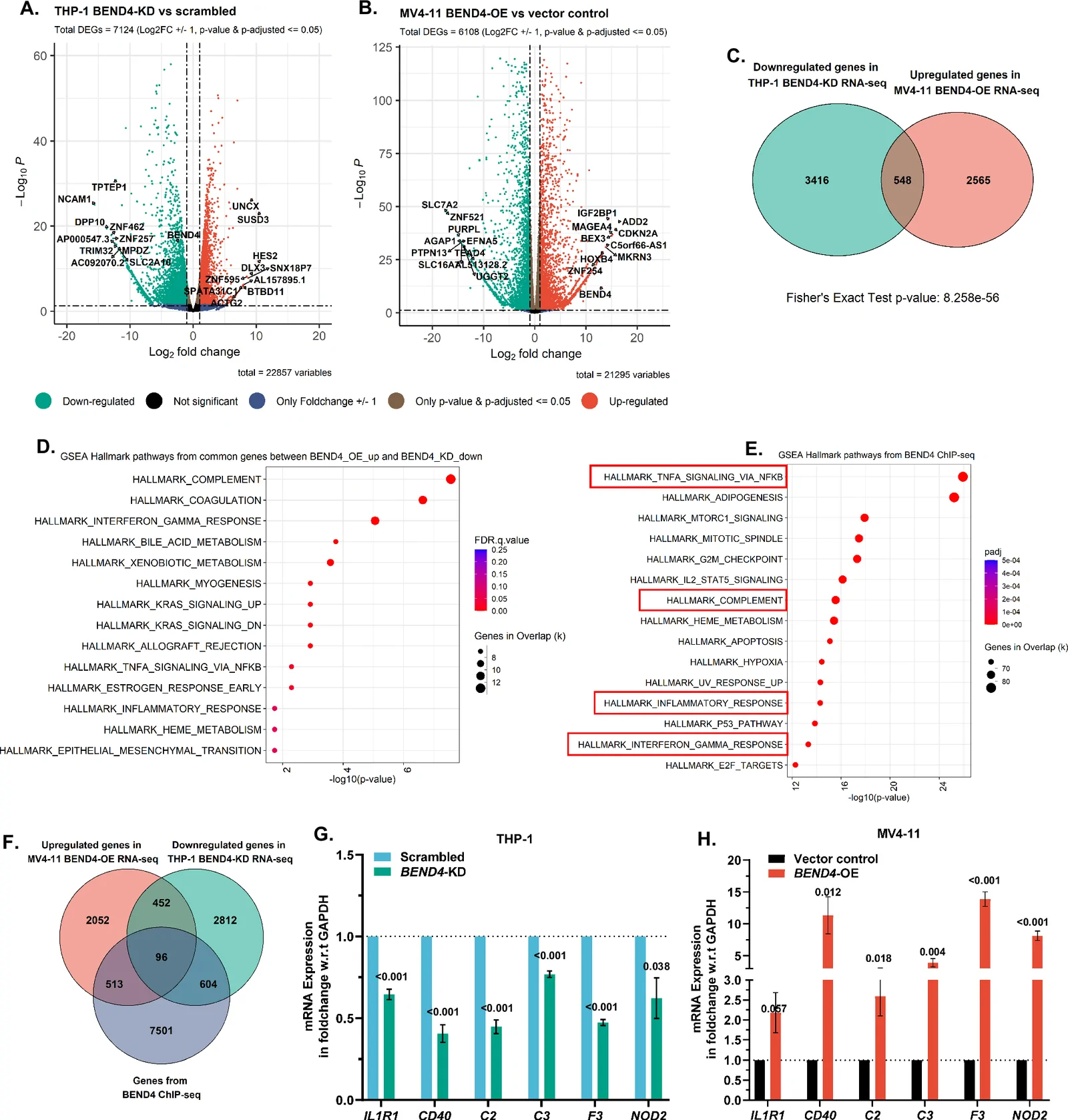
Transcriptomic profiling and chromatin occupancy of BEND4 reveal its role in regulating inflammatory and oncogenic gene programs in AML cells. **A** Volcano plot showing differentially expressed genes (DEGs) from RNA-seq analysis of THP-1 BEND4-KD versus THP-1 scrambled control cells. A total of 7124 DEGs were identified (Log2 fold change ±1; *p*-value and adjusted *p*-value ≤ 0.05; total variables = 22,857). Downregulated genes are shown in green, upregulated genes in red, genes meeting only the fold change threshold in yellow, genes meeting only the *p*-value and adjusted *p*-value threshold in blue, and non-significant genes in gray. The top 10 upregulated and downregulated genes (along with *BEND4*) are labeled. **B** Volcano plot showing DEGs from RNA-seq analysis of MV4-11 BEND4-OE versus MV4-11 vector control cells. A total of 6108 DEGs were identified (Log2 fold change ±1; *p*-value and adjusted *p*-value ≤ 0.05; total variables = 21,295). Color coding as described in (**A**). The top 10 upregulated and downregulated genes are labeled. **C** Venn diagram showing the overlap between genes downregulated in THP-1 BEND4-KD RNA-seq and genes upregulated in MV4-11 BEND4-OE RNA-seq, identifying 548 common genes representing high-confidence BEND4-regulated targets. Statistical significance of the overlap was determined by Fisher’s Exact Test. **D** Gene Set Enrichment Analysis (GSEA) dot plot showing enriched MSigDB Hallmark pathways from the 548 common genes identified in (**C**). Dot size represents the number of genes in overlap (k), and dot color represents the FDR *q*-value. Pathways are ranked by −log10(*p*-value). **E** GSEA dot plot showing enriched MSigDB Hallmark pathways from BEND4 ChIP-seq target genes in MV4-11 BEND4-OE cells. Dot size represents the number of genes in overlap (k), and dot color represents the FDR q-value. Pathways are ranked by −log10(*p*-value). Red boxes highlight pathways convergent with RNA-seq analysis—TNF-α signaling via NF-κB, complement, and IFN-γ response. **F** Three-way Venn diagram showing the overlap between upregulated genes in MV4-11 BEND4-OE RNA-seq (*n* = 513 shared with only ChIP-seq), downregulated genes in THP-1 BEND4-KD RNA-seq (*n* = 604 shared with only ChIP-seq), identifying 96 direct transcriptional targets of BEND4 at the intersection of all three datasets. **G** RT-qPCR validation of selected BEND4 target genes (*IL1R1*, *CD40*, *C2*, *C3*, *F3*, and *NOD2*) in THP-1 BEND4-KD cells compared to scrambled control cells. mRNA expression is shown as fold change relative to *GAPDH*. Data represent the mean ± SD of three independent experiments. *p*-values were calculated using multiple t-tests. **H** RT-qPCR validation of selected BEND4 target genes (*IL1R1*, *CD40*, *C2*, *C3*, *F3*, and *NOD2*) in MV4-11 BEND4-OE cells compared to vector control cells. mRNA expression is shown as fold change relative to *GAPDH*. Data represent the mean ± SD of three independent experiments. *p*-values were calculated using multiple t-tests.

To gain further molecular insight into the mechanisms underlying BEND4-mediated chemoresistance and aggressive cellular phenotype, we performed RNA sequencing of AML cells with BEND4 overexpression (MV4-11 BEND4-OE), BEND4 knockdown (THP-1 BEND4-KD), and corresponding empty vector controls. Transcriptome analysis identified a total of 6108 and 7124 differentially expressed genes (Log2Foldchange ± 1, *P*-value and adjusted *P*-value ≤ 0.05) in BEND4 overexpression and knockdown, respectively, out of which 3113 genes were upregulated in BEND4 overexpression, while 3964 genes were downregulated in BEND4 knockdown (Fig. 5A, B). To identify genes likely regulated by BEND4 with high confidence, we intersected genes upregulated in MV4-11 BEND4-OE with genes downregulated in THP-1 BEND4-KD, identifying 548 common genes (Fig. 5C). Pathway enrichment analysis of the common genes using GSEA showed enrichment of multiple pro-tumorigenic pathways encompassing inflammatory and immune signaling, oncogenic signaling, metabolic reprogramming, and tissue remodeling, several of which have established roles in AML progression and poor prognosis (Fig. 5D)^38–45^. Notably, pathway enrichment analysis of upregulated genes from relapsed and refractory AML patients in the BEAT-AML and GSE165430 datasets revealed enrichment of overlapping tumor-promoting signaling pathways (Fig. S12B, C), suggesting that BEND4-regulated transcriptional programs may be relevant to AML disease progression.

Considering the predicted and reported roles of BEN-domain proteins in chromatin organization and transcriptional regulation^46–48^, we performed ChIP-seq in MV4-11 BEND4-OE cells to determine whether BEND4 directly occupies regulatory regions of these pathways. Indeed, ChIP-seq profiling confirmed strong BEND4 enrichment around peak summits relative to input (Fig. S13A), with read density sharply centered at peak summits across all called peaks (Fig. S13B), and predominant binding at promoter-proximal regions (Fig. S13C), indicating a primary role in promoter-associated transcriptional regulation. To identify the functional programs associated with BEND4 chromatin occupancy, we performed GSEA on genes annotated to BEND4-bound promoter-proximal peaks (±3 kb from TSS). This revealed enrichment of multiple hallmark pathways, including inflammatory and immune signaling, cell cycle regulation and proliferation, apoptosis, DNA damage response, and metabolic reprogramming (Fig. 5E). Importantly, TNF-α signaling via NF-κB^42^, IFN-γ response^40^, complement^38,39^, and inflammatory response^43^ emerged as the pathways shared between ChIP-seq and RNA-seq analyses (Fig. 5E). This overlap strongly supports a direct role for BEND4 in regulating tumor-associated pathways in AML cells.

To identify genes directly regulated by BEND4, we integrated ChIP-seq targets with RNA-seq differentially expressed genes and identified 96 overlapping genes as potential direct transcriptional targets of BEND4 (Fig. 5F). GSEA of these 96 genes confirmed enrichment of the same four pathways (Fig. S13D), supporting the specificity of BEND4-mediated regulation. Among the key contributor genes to these four shared pathways, *IL1R1*, *CD40*, *C2*, *C3*, *F3*, *NOD2*, *CD80*, and *CCL2* were identified as the top overlapping genes across TNF-α/NF-κB, IFN-γ response, complement, and inflammatory response pathways, and significant BEND4 binding was confirmed at their regulatory regions (Fig. S14A–H and Table S3). To validate direct transcriptional regulation, we examined the expression of *IL1R1*, *CD40*, *C2*, *C3*, *F3*, and *NOD2* by RT-qPCR in BEND4-perturbed AML cells. Consistent with the expression pattern of *BEND4*, these genes were significantly downregulated in THP-1 BEND4-KD cells (Fig. 5G) and upregulated in MV4-11 BEND4-OE cells (Fig. 5H), confirming their direct transcriptional regulation by BEND4. Together, these results demonstrate that BEND4 directly occupies promoter and regulatory regions of key inflammatory and pro-tumorigenic signaling genes and transcriptionally regulates components of the TNF-α/NF-κB, IFN-γ, complement, and inflammatory response pathways in AML cells. These findings establish a direct mechanistic link between BEND4’s chromatin-regulatory function and the inflammatory and oncogenic gene regulatory networks associated with AML progression.

### BEND4 overexpression leads to higher tumorigenicity and poor survival in mice

**Fig. 6:**
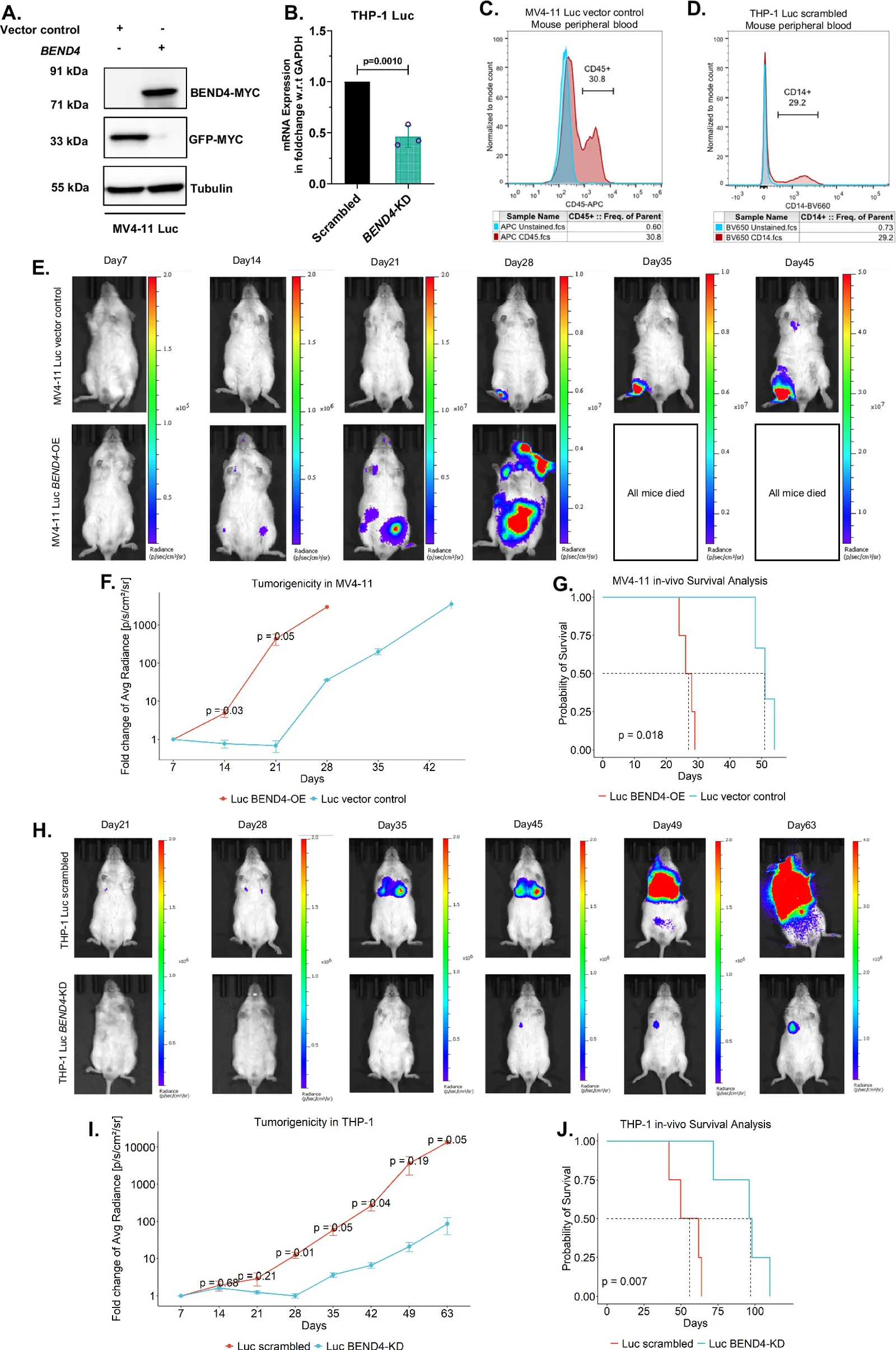
BEND4 regulates leukemia progression and survival in in vivo AML xenograft models. **A** Cell lysates were prepared from MV4-11 Luc cells stably expressing BEND4 with MYC- Flag tag or the vector control expressing GFP with MYC-Flag tag. Western blot using an anti- MYC antibody identifies bands of appropriate molecular weight in each lane. **B**. Bar plot for the RT-qPCR of mRNA level of *BEND4* from the THP-1 Luc cells with shRNA- mediated BEND4 knockdown compared to THP-1 Luc scrambled cells. The values are in fold- change with respect to GAPDH. Data represent the mean ± SEM of 3 independent experiments. The *p*-value was calculated using t-test. **C**, **D** Histogram of flow cytometry analysis from mouse peripheral blood of CD45-positive MV4-11 Luc vector control and CD14-positive THP-1 Luc scrambled cells. The blue histogram represents the unstained population, and the red histogram represents the CD45 or CD14 positive population of cells. **E** Representative bioluminescent images of mice injected with either MV4-11 Luc BEND4- OE cells (*n* = 4) or MV4-11 Luc vector control cells (*n* = 3). **F** Tumor progression was quantified as luciferase activity intensity from bioluminescence imaging (BLI) at the indicated time and plotted as fold change of Avg Radiance [p/s/cm²/sr] with respect to week 1 (Day 7) values for each animal with MV4-11 Luc BEND4 overexpressing cells or MV4-11 Luc vector control cells. The *p*-values were calculated using multiple t-test. **G** Kaplan–Meier plots showing the overall survival of mice bearing either MV4-11 Luc BEND4-OE cells (*n* = 4) or MV4-11 Luc vector control cells (*n* = 3). The *p*-value was obtained from the log-rank test. **H** Representative bioluminescence images of mice injected with either THP-1 Luc scrambled cells (*n* = 4) or THP-1 Luc BEND4-KD cells (*n* = 4). **I** Tumor progression was quantified as luciferase activity intensity from bioluminescence imaging (BLI) at the indicated time and plotted as fold change of Avg Radiance [p/s/cm²/sr] with respect to week 1 (Day 7) values for each animal injected with either THP-1 Luc scrambled cells (*n* = 4) or THP-1 Luc shBEND4 cells (*n* = 4). The *p*-values were calculated using multiple t-test. **J** Kaplan–Meier plots showing the overall survival of mice bearing either THP-1 Luc scrambled cells (*n* = 4) or THP-1 Luc BEND4-KD cells (*n* = 4). The *p*-value was obtained from the log-rank test. Results in each line graph are the composite data from at least three independent experiments (mean ± SD).

To further study the role of BEND4 in tumorigenicity and survival in vivo, we established an AML mouse model. First, we stably expressed luciferase in AML cell lines (MV4-11 Luc, THP-1 Luc). Subsequently, we overexpressed BEND4 in MV4-11 Luc (MV4-11 Luc BEND4-OE) cells and knocked down BEND4 in THP-1 Luc cells (THP-1 Luc BEND4-KD). The over-expression and knockdown of BEND4 were confirmed at protein and transcript levels (Fig. 6A, B). MV4-11 Luc BEND4-OE and THP-1 Luc BEND4-KD and their respective vector control cells were injected into NOD-SCID mice via tail-vein injection. Engraftment of the AML cells was confirmed by flow cytometry analysis of human CD45-positive MV4-11 Luc cells (Fig. 6C) and human CD14-positive THP-1 Luc cells (Fig. 6D) in the mouse peripheral blood collected after 42 days of injection for MV4-11 cells and 56 days for THP-1 cells. AML progression in the mice was monitored using bioluminescence imaging using the IVIS Spectrum imaging system. We observed that mice injected with MV4-11 Luc BEND4-OE cells had increased bioluminescence compared to the vector control (Fig. 6E, F), while a lesser tumor progression was observed in THP-1 Luc *BEND4*-KD cells compared to the vector control (Fig. 6H, I). Furthermore, *BEND4* overexpression significantly reduced (1.9-fold) overall survival in mice to 27 days compared to 51 days in vector control (Fig. 6G); conversely, *BEND4* knockdown significantly increased (1.7-fold) the overall survival to 97 days compared to 56 days of vector control (Fig. 6J). These in vivo findings confirmed that BEND4 overexpression increased tumorigenicity and poor survival in AML, whereas its knockdown had the opposite effect. Taken together, these data strongly support BEND4 as a critical regulator of AML progression and survival.

## Discussion

AML prognostication and risk stratification are highly dominated by mutation and chromosomal aberration-based markers^49,50^. While these biomarkers serve the purpose of cytogenetic risk classification and guidance to the therapeutic intervention faithfully, they are often challenged by the lack of any cytogenetic abnormalities or mixed cytogenetic abnormalities, or they cannot serve as a therapeutic target^21^. To overcome those challenges, an alternative approach is to study gene expression-based biomarkers that are also more amenable to therapeutic targeting^51^. Although there are a few studies done in the past decade, they are hampered by two major limitations: either the identified genes were too large in number to be successfully implemented in clinical practice^52^, or the prognostic associations reported for single gene expression-based markers were largely correlative and lacked comprehensive functional validation to establish a cause-and-consequence relationship^53,54^.

In this study, our in-silico analysis of the transcriptome of multiple large cohorts of AML patients and its validation in independent cohorts using both in-silico and orthologous techniques collectively identified high expression of a novel BEN-domain containing protein 4, BEND4, in de novo AML patients of adverse prognosis and relapsed or refractory AML patients. BEND4 belongs to the family of BEN domain-containing proteins, and its overexpression and high copy number have been linked to metastasis and increased cancer risk in multiple cancers^55,56^. Our study also showed that *BEND4* overexpression not only leads to significant poor overall and relapse-free survival, but high *BEND4* expression is an independent risk factor in AML. Moreover, high *BEND4* expression in the conventional favorable risk group patients, harboring either functional *FLT3* or mutated *NPM1*, can lead to poor survival. Notably, comparison with the ELN risk classification demonstrated modest complementary prognostic value when *BEND4* expression was combined with ELN, while addition of *BEND4* expression to the LSC17^57^ transcriptomic classifier did not yield meaningful improvement across cohorts (data not shown), likely reflecting the biological overlap between BEND4-regulated transcriptional programs and the LSC17 stemness signature. Importantly, ROC analysis of *BEND4* mRNA expression from RT-qPCR data identified a ΔCt threshold of <12.75 as a preliminary cutoff, supporting the potential utility of *BEND4* as a simple, cost-effective, single gene expression-based prognostic marker for stratifying adverse and favorable cytogenetic risk in AML; however, validation of this threshold in larger independent cohorts will be critical for establishing its definitive clinical utility. At the functional level, overexpression of BEND4 in vitro increased resistance to chemotherapeutics and promoted AML progression, while BEND4 knockdown significantly heightened sensitivity to those drugs and impeded AML progression. Furthermore, the transcriptome of BEND4 perturbation revealed an expected abundance of deregulated genes, given that BEND4 is predicted to have a function in chromatin organization and transcription regulation^46–48^. Consistent with this, our ChIP-seq analysis confirmed predominant but not exclusive promoter-proximal BEND4 binding (~ 35.7% within 1 kb of TSS), with additional occupancy at intronic and distal intergenic regulatory regions, and integration with RNA-seq data identified 96 high-confidence direct transcriptional targets. These findings provide mechanistic evidence that BEND4 functions as a chromatin-associated transcriptional regulator of key inflammatory and oncogenic gene programs in AML cells, including TNF-α/NF-κB signaling, the complement pathway, and the IFN-γ response. Corroborating this, subsequent RT-qPCR validation confirmed that BEND4 directly regulates key genes from these pathways, including *IL1R1*, *CD40*, *C2*, *C3*, *F3*, and *NOD2*, several of which have been previously reported to play roles in AML progression and poor prognosis^39,41,58–60^. Finally, our AML mouse model with BEND4 overexpressing cells showed increased tumor burden and poor survival, whereas BEND4 knocked down cells showed reduced tumor burden and better survival. These in vitro and in vivo studies collectively demonstrate that *BEND4* is not merely a single gene expression-based prognostic marker but also a functional driver of AML progression and therapy resistance, thereby addressing the key limitation of previously reported single gene expression-based markers that lacked functional validation. Together, these findings establish BEND4 as a candidate therapeutic target in AML. While the absence of specific inhibitors targeting the BEND4 protein or the BEN domain currently precludes direct therapeutic intervention, the identification of druggable downstream effectors and inflammatory signaling pathways regulated by *BEND4* represents a compelling avenue for future pharmacological investigation aimed at limiting AML progression.

In summary, this study identifies *BEND4* as a robust single gene expression-based prognostic marker capable of identifying adverse prognosis with the prospect for clinical implementation. Functional validation further established a novel role for BEND4 as a key driver of AML progression and chemoresistance, highlighting its candidacy as a therapeutic target in AML.

## Methods

### Transcriptome data

HTSeq count data for TCGA-LAML and BeatAML were downloaded from the GDC data portal (https://portal.gdc.cancer.gov/). Microarray raw data, i.e., CEL files for GSE10358 and GSE14468 datasets, were downloaded from the NCBI Gene Expression Omnibus (https://www.ncbi.nlm.nih.gov/geo/). Raw FASTQ files for RNA-Sequencing datasets GSE103424 and GSE83533 were downloaded from NCBI GEO and NCBI dbGaP (https://dbgap.ncbi.nlm.nih.gov/) after due permission from the authors, respectively. Lastly, for the GSE165430 dataset, the processed HTseq count data were downloaded from NCBI GEO.

### Microarray data processing

Microarray datasets used the Affymetrix Human Genome U133 Plus 2.0 Array platform. The raw CEL files were processed and RMA normalized using arrays and the affy R/Bioconductor packages. After normalization, samples were annotated using GPL570 annotation in RStudio.

### RNA-seq

RNA was isolated using Qiagen RNeasy Kit (Cat no. 74104) according to the manufacturer’s protocol. Further steps were outsourced to the NGS service provider miBiome Therapeutics LLP (Mumbai, India) and Nucleome Informatics Private Limited (Hyderabad, India). Briefly, the RNA samples were quantified on a Qubit fluorometer, and appropriate dilutions were loaded on a high-sensitivity RNA screen tape to determine the RNA integrity number (RIN). Samples with 28S/18S peak integrated area ratios >2 were used for library preparation. The NEBNext Ultra II Directional RNA Library Prep Kit for Illumina (cat. no. E7760L) was used for library preparation. The cleaned libraries were quantified on a Qubit fluorometer and validated on a high-sensitivity D1000 screen tape. Prepared libraries were sequenced on an Illumina NovaSeq 6000 sequencer to generate 2 × 150 bp reads per sample.

### RNAseq data processing and differential gene expression analysis

Quality control for downloaded FASTQ files, as well as our cell line transcriptome data, was initially performed using the FastQC tool (v0.11.8). If necessary, adapter trimming was done using Trimmomatic (v0.39), followed by a second round of QC. Samples that passed QC were aligned to the latest reference human genome (GRCh38.p13), downloaded from the GENCODE website, and annotated using the corresponding GTF file (GENCODE v38) with the STAR aligner (v2.7.0c). The bam files generated by the STAR aligner were quantified, and subsequent raw read-counts files were generated using the HT-Seq tool (v0.11.1). Finally, the read-counts were used for differential gene expression using the DESeq2 package in R software (RStudio v2024.12.0).

### Cell culture

Human AML cell lines THP-1, HL-60, and KG1a were purchased from the Cell repository of the National Centre for Cell Science (NCCS, India); MOLM13, OCI-AML2, and MV4-11 cell lines were kind gifts from Dr. Syed K. Hasan, TMC-ACTREC, India. THP-1, HL-60, MV4-11, and MOLM13 cells were grown in RPMI 1640 (Gibco, Catalog no. 31800022) supplemented with 10% FBS (Gibco, Catalog no. A56707-01) and 1× antibiotic-antimycotic solution (Himedia, A002). KG1a cells were grown in IMDM (Gibco, Catalog no. 12200036) supplemented with 20% FBS and 1× antibiotic-antimycotic solution. OCI-AML2 cells were grown in 90% alpha-MEM (Gibco, Catalog no. 11900024) (with ribo- and deoxyribonucleosides) supplemented with 10% FBS. HEK-293FT cells (a kind gift from Dr. Amit Dutt, TMC-ACTREC, India) were grown in DMEM (Gibco, Catalog no. 12100046) supplemented with 10% FBS and 1× antibiotic-antimycotic solution. Cells were maintained in a humidified CO2 incubator at 37 °C.

### Plasmids

For BEND4 overexpression, pLenti-c-Myc-DDK-P2A-Puro-BEND4 (Origene, CAT: RC212072L3) was used, and pLenti-c-MYC-DDK-Puro-GFP (Addgene, 123299) or pLenti-C-Myc-DDK-P2A-Puro (Origene, CAT: PS100092) were used as vector control. For BEND4 knockdown, 3 different shRNAs with TRC clone ID TRCN0000245693, TRCN0000245694, and TRCN0000245695 were purchased from Sigma and pLKO.1-puro Empty Vector Control (Sigma, #SHC001) was used as vector control. For stable luciferase expression, Lenti-luciferase-P2A-Neo plasmid (Addgene #105621) was used. These vectors, along with pMD2.G (Addgene, 12259) and psPAX2 (Addgene, 12260), were co-transfected into HEK-293FT cells using Lipofectamine 3000 transfection reagent (Invitrogen, L3000001) to produce lentiviral particles. After 2 days of transduction using the Spinoculation protocol^61^ with said viral particles, cells were selected by either puromycin (Himedia, CMS8861) for 5-7 days with a concentration of 1 µg/mL for MOLM13, 2 µg/mL for both THP-1 and MV-4-11; or G418 (Himedia, TC025) of concentration 500 ng/mL for 10–14 days for both THP-1 and MV-4-11.

### Immunoblotting

Cell lysates were prepared in RIPA lysis buffer with 1x protease inhibitor cocktail (Thermo Scientific, 78437), and protein was quantified using the BCA protein assay kit (Thermo Scientific, 23225). Fifty micrograms of protein were loaded and resolved on SDS-PAGE and transferred to a nitrocellulose membrane. These membranes were probed with MYC-tag (Cell Signaling Technology, 2276S) for BEND4-MYC-FLAG protein, actin (Cell Signaling Technology, 3700S), and beta-Tubulin (Cell Signaling Technology, 86298S) primary antibodies (1:4000 dilution). Blots were developed using ECL reagent (BioRad, #1705061) and a BioRad Chemidoc machine.

### Immunohistochemistry

FFPE blocks of bone marrow biopsies of AML patients were obtained after ethics committee approval. IHC followed by immunofluorescence (IHC-IF) was done as described previously^62^. Briefly, sections from the FFPE blocks were fixed on poly-l-lysine-coated glass slides and rehydrated. After water washing, antigen retrieval was done by boiling the slides in Tris-EDTA buffer (pH = 9) for 15 min. Slides were then washed using TBS-Tween buffer and blocked with 1% horse serum in PBS for 30 min at room temperature. Sections were incubated overnight at 4 °C with anti-BEND4 antibody (Sigma, HPA036850) at a 1:100 dilution. After PBS wash, cells were incubated with a secondary antibody with Alexa Fluor 633 (Invitrogen, catalog #A-21070) at a 1:300 dilution. Cells were washed with PBS again and then mounted with VECTASHIELD mounting medium (Vector Laboratories, H-1000-10) containing 0.01 µg/µL DAPI (Sigma-Aldrich, D9542). Imaging was done using a Zeiss LSM 780 Confocal Microscope, and its quantification was done using ImageJ software.

### RT-qPCR

RNA was isolated using Qiagen RNeasy Kit (Cat no. 74104) according to the manufacturer’s protocol. cDNA preparation and qPCR were done as described previously^62^. GAPDH or RPL19 was used as the internal control. Oligonucleotide details are provided in Table S4.

### Cell viability and cell proliferation assay

1 × 10^4^ cells were either treated with doxorubicin (Fresenius Kabi) and cytarabine (Biobin) at different concentrations for 48 h, followed by an MTT assay for cell viability. A detailed protocol for the MTT assay is described previously^62^. For the cell proliferation assay, an MTT assay was done after seeding 1 × 10^4^ cells in 0 h, 24 h, 48 h, and 72 h. Cell proliferation was calculated using the formula: Relative cell proliferation (%) = (Aₓh/A₀h) × 100, Where A₀h = absorbance at 0 h and Aₓh = absorbance at time *x* (24, 48, and 72 h).

### Clonogenic assay

The protocol was followed as published^63^. Briefly, 1 × 10^3^ cells were seeded directly in 1 mL of methyl cellulose agar (1.6%) with 1 mL of 2X RPMI supplemented with 20% FBS and 2X antibiotic-antimycotic solution in a 1:1 ratio. Colonies were imaged using Incucyte® Live-Cell Analysis System and counted using ImageJ after 12–14 days. Number of colonies, mean colony area, colony-forming efficiency (CFE), colony size distribution, median colony area, and total integrated colony area were calculated and plotted using GraphPad and R Studio.

### Chromatin immunoprecipitation followed by sequencing (ChIP-seq)

Chromatin immunoprecipitation followed by sequencing (ChIP-seq) was performed to identify genomic regions associated with BEND4. MV4-11 cells stably expressing BEND4-Myc or an empty vector control (GFP-Myc) were cultured under standard conditions. Chromatin immunoprecipitation was carried out using the SimpleChIP® Enzymatic Chromatin IP Kit (Cell Signaling Technology, #9003) according to the manufacturer’s protocol. Briefly, approximately 4 × 10^7^ cells per immunoprecipitation were crosslinked with 1% formaldehyde for 10 min at room temperature, and the reaction was quenched with glycine. Cells were washed with ice-cold PBS containing protease inhibitors, and nuclei were isolated using buffers provided in the kit. Chromatin was fragmented by micrococcal nuclease digestion followed by brief sonication to generate DNA fragments predominantly in the 150–900bp range. Immunoprecipitation was performed overnight at 4 °C using anti-Myc antibody (Cell Signaling Technology, #2276S) to capture Myc-tagged chromatin complexes. Since both the BEND4 overexpression construct and the empty vector control carry a Myc tag (BEND4-Myc and GFP-Myc), the same antibody was used for immunoprecipitation. Antibody–chromatin complexes were collected using Protein G magnetic beads supplied with the kit, washed sequentially with low-salt and high-salt buffers, and eluted. Crosslinks were reversed at 65 °C followed by Proteinase K digestion, and DNA was purified using spin columns provided in the kit. Library preparation and sequencing were performed by miBiome Therapeutics LLP (Mumbai, India). ChIP-seq libraries were constructed using the NEBNext Ultra II DNA Library Prep Kit for Illumina following the manufacturer’s protocol. Libraries were sequenced on the Illumina NovaSeq 6000 platform to generate approximately 40 million paired-end reads per sample (150 bp read length). Raw sequencing reads were trimmed using fastp and aligned to the GRCh38 human reference genome using BWA. Alignments were processed with SAMtools to remove low-quality reads (MAPQ < 30), secondary and supplementary alignments, and reads overlapping ENCODE blacklist regions, followed by duplicate marking. Biological replicates of BEND4 ChIP samples were merged for downstream analysis. Peak calling was performed using MACS3 with paired-end mode and matched input control using a false discovery rate threshold of *q* < 0.05. Genome-wide normalized coverage tracks were generated using deepTools. Enrichment around transcription start sites was analyzed using deepTools, and peaks were annotated to nearby genes using ChIPseeker R package and genes were assigned to peaks based on promoter-proximal regions (within ±3 kb of TSS). The annotated promoter-proximal gene list was subsequently used for GSEA Hallmark pathway enrichment analysis using fgsea R package.

### AML mouse model

NOD.Cg-Prkdc^scid^/J (NOD-SCID) mice used in this study were obtained from The Jackson Laboratory, USA, through the in-house Laboratory Animal Facility (LAF) (registration no. 65/GO/ReiBiBt/S/1999/CPCSEA). All breeding and maintenance of animals in the LAF were carried out as per the guidelines approved by the Committee for the Purpose of Control and Supervision of Experiments on Animals, Government of India (CPCSEA). Mice were housed under standard conditions with ad libitum access to food and water. All animal experiments and procedures performed in this study were approved by the Institutional Animal Ethics Committee (IAEC) (Project no. 06/2024) and the Institutional Biosafety Committee (Project no. TMC/IBSC/Apr 2024/7). All experiments performed on live vertebrates were in accordance with relevant guidelines and regulations.

Stable luciferase-expressing THP-1 Luc and MV4-11-Luc cells were checked for luciferase activity in a 96-well plate using 3 mg/mL D-luciferin potassium salt (Biosynth AG, #L-8220). BEND4 was stably overexpressed in MV4-11 Luc cells and knocked down in THP-1-Luc cells, along with their respective control vectors. 2 × 10^6^ cells suspended in 100 µl RPMI 1640 media without FBS were then injected into male, 6–8 weeks old NOD-SCID mice by tail-vein after 24 h of 20 mg/kg busulfan (Bucelon, CELON Labs) intraperitoneal treatment. Mice were randomly assigned to experimental groups based on body weight at the start of the study. For monitoring tumor progression, mice were anesthetized using isoflurane (Baxter India Pvt. Ltd., catalog no. IN2L9101) combined with oxygen, followed by injection of 150 mg/kg D-luciferin potassium salt intraperitoneally, and bioluminescence imaging was done using IVIS Spectrum-CT (Perkin Elmer). Post luciferin administration, intensity values were calculated using Living Image software (Perkin Elmer), and the fold changes in Avg Radiance values expressed in [p/s/cm²/sr] were plotted. Blinding was not applied during cell injection and in vivo monitoring, as group identity was operationally required during these procedures; however, all data analysis was performed using objective quantitative measurements of bioluminescence signal intensity using Living Image software (Perkin Elmer), thereby reducing the potential for subjective bias. Animals were monitored regularly for leukemia-associated symptoms, and humane endpoints were defined by the development of severe disease signs, including hind-limb paralysis or persistent diarrhea. At study end or upon reaching humane endpoints, mice were euthanized by carbon dioxide (CO_2_) inhalation followed by confirmation of death by a secondary physical method.

### Apoptosis assay

Doxorubicin and cytarabine (Ara-C) drug-treated cells were stained with Annexin V tagged with FITC fluorochrome and 7-Amino-Actinomycin D (7-AAD) according to the protocol mentioned in the Annexin V-FITC Early Apoptosis Detection Kit (Cell Signaling Technology, 6592) and BD Pharmingen™ 7-AAD (catalog No: 559925). Briefly, 1 × 10^5^ cells were harvested after 48 h of drug treatment, washed with cold 1X PBS and resuspended in 100 µl 1× Annexin V Binding Buffer. Cells were then stained with 2 µl annexin V FITC stain and 2 µl 7-AAD for 20 min on ice and analyzed by flow cytometry (Thermo-Fisher, Attune NxT Flow Cytometer) using gates and compensation designed to exclude debris, compensate for doxorubicin autofluorescence and include FITC and 7-AAD positive stain compared with unstained controls. The acquired data were analyzed using FlowJo (v10).

### Cell cycle analysis

Cells were harvested and resuspended in ice-cold 300 µl 1× PBS. 700 µl chilled ethanol (final concentration 70% v/v) was added dropwise and incubated −20 °C for overnight after gentle mixing for fixation. Pellets of fixed cells were washed once in ice-cold 1X PBS and passed through 70 μm cell strainer before resuspending in propidium iodide staining buffer (PBS with 100 μg/ml RNase A, 50 μg/ml propidium iodide and 0.1% Triton-X100). Cells were then incubated on ice in dark for 20 min before acquisition by flow cytometry (Thermo-Fisher, Attune NxT Flow Cytometer). The acquired file was analyzed using Modfit LT v5 software.

### Flow cytometry of mice peripheral blood

Mouse peripheral blood was collected from the retro-orbital sinus. After RBC lysis using RBC lysis buffer (BioLegend #420301), cells were washed twice using 1× PBS and incubated with human CD14-BV650 (BD Horizon™ #563419) or human CD45-APC (BD Horizon™ #560973) according to the manufacturer’s protocol. Flow cytometry analysis was done using BD FACSAria™ III and Thermo-Fisher Attune NxT Flow Cytometer, and data were analyzed using FlowJo (v10).

### Statistics and replicates

The Z-score matrix was calculated using the formula *Z* = (*x*-*µ*)/*σ* in RStudio. All statistical tests mentioned in text or figure captions were performed using either RStudio v2024.12.0 or GraphPad Prism v8 using the tests described for each experiment. Data from three independent biological replicates were considered unless stated otherwise for the statistical test.

#### **Ethics approval and consent to participate**

All research involving human participants, material, and data was performed in accordance with the Declaration of Helsinki. Bone marrow biopsies (FFPE blocks) were acquired from the Pathology Department, Tata Memorial Hospital, retrospectively after approval by the Institutional Ethics Committee (TMC-IEC III, DCGI registration number: ECR/149/Inst/MH/2013/RR-24). Written informed consent was waived by the committee for retrospective FFPE block collections. Blood samples from AML patients were accrued after approval by the Institutional Ethics Committee (TMC-IEC III, DCGI registration number: ECR/149/Inst/MH/2013/RR-24). Written informed consent was obtained from all patients in a language understood by them.

## Supplementary information

Supplementary Figures.

Supplementary Data 1.

## Data Availability

The RNA sequencing and ChIP sequencing data generated for the current study are available in the EMBL-EBI ArrayExpress repository, under accession numbers E-MTAB-15162 and E-MTAB-16748, respectively.
