# Supplementary material for: Discovery of BEND4 as a novel single-gene prognostic marker and therapeutic target for adverse AML
Source: NPJ Precis Oncol. 2026 Jun 6;10:351. doi: 10.1038/s41698-026-01534-7 (PMC13575146; doi:10.1038/s41698-026-01534-7)

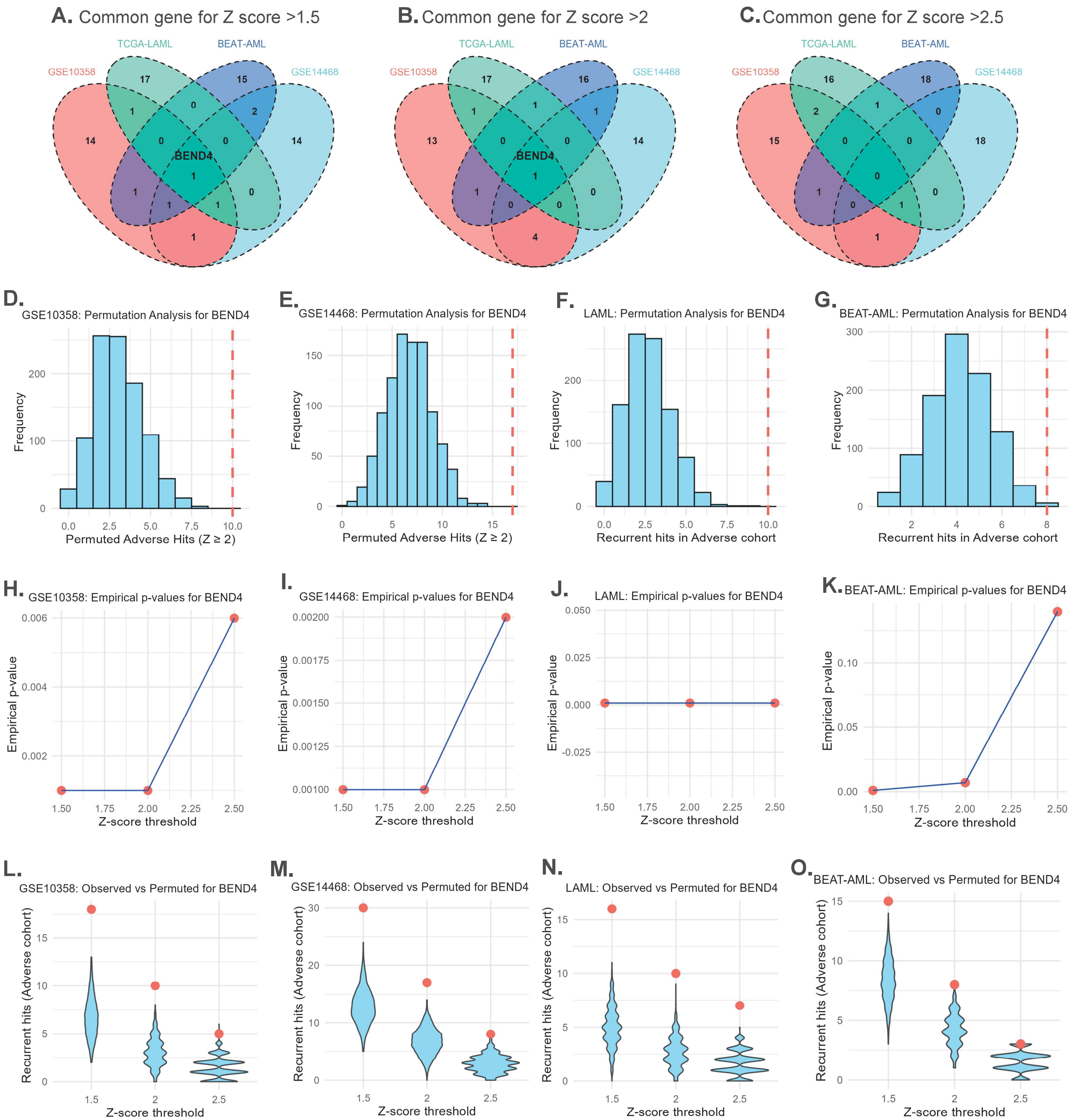

Figure S1: Robustness analysis of the Z-score–based gene prioritization strategy identifying BEND4.

(A–C) Venn diagrams showing genes recurrently upregulated in adverse-risk AML patients across four independent datasets (GSE10358, GSE14468, TCGA-LAML, and BEAT-AML) using different Z-score thresholds: Z > 1.5 **(A)**, Z > 2 **(B)**, and Z > 2.5 **(C)**. *BEND4* was the only gene consistently shared across all four cohorts at Z > 1.5 and Z > 2 thresholds. At Z > 2.5, no gene was found to be common across all four cohorts, reflecting the increasingly stringent nature of the threshold; however, *BEND4* remained among the top recurrently upregulated genes in three of four cohorts, further supporting the robustness of its identification across a range of thresholds. **(D–G)** Permutation analysis evaluating the significance of *BEND4* recurrence in the adverse-risk cohort (Left to Right: GSE10358, GSE14468, TCGA-LAML, and BEAT-AML). Histograms show the distribution of recurrent hits obtained from random permutations of patient labels, with the observed recurrence for *BEND4* indicated by the red dashed line.

(H–K) Empirical p-values for *BEND4* recurrence calculated across different Z-score thresholds (1.5–2.5) for each dataset (Left to Right: GSE10358, GSE14468, TCGA-LAML, and BEAT-AML).

(L-O) Comparison of observed versus permuted recurrence frequencies for *BEND4* in the adverse-risk cohort across Z-score thresholds (1.5, 2.0, and 2.5) for each dataset (Left to Right: GSE10358, GSE14468, TCGA-LAML, and BEAT-AML). The y-axis represents the number of recurrent hits in the adverse cohort. Red points represent the observed recurrence frequency for *BEND4* at each threshold, while violin plots represent the distribution of recurrence frequencies obtained from permutation simulations.

A.

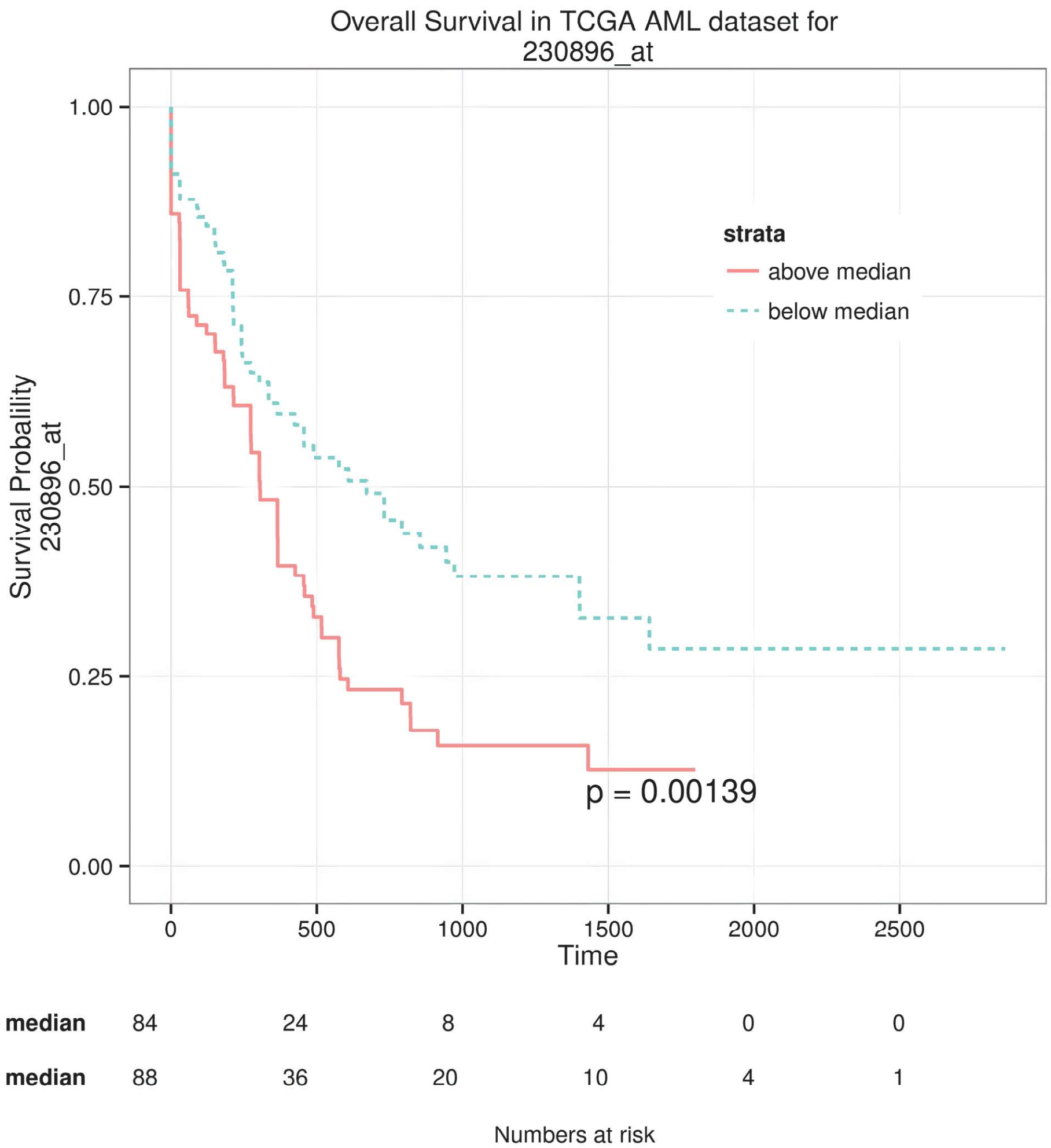

Figure S2: High *BEND4* associates with poor survival

A. Overall survival analysis from BloodSpot 3.0 database of AML patients with *BEND4* high and low expression. The P values were obtained by the log-rank test for comparison of OS.

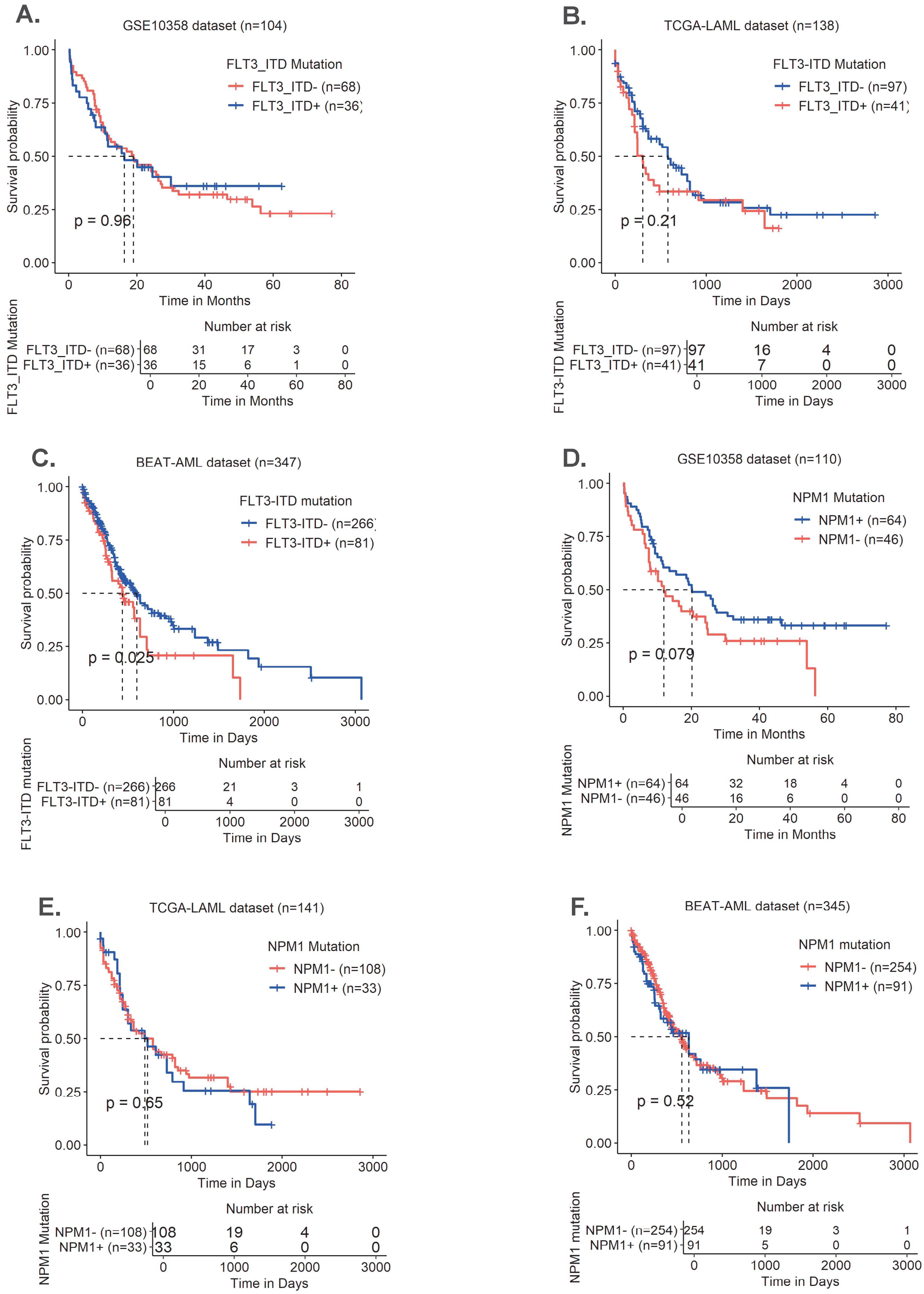

Figure S3: *FLT3*-ITD and *NPM1* mutations stratify AML patients

A-C. Kaplan Meier curve for overall survival (OS) of patients according to *FLT3*-ITD mutation positive or negative patients from GSE10358, TCGA-LAML and BEAT-AML datasets. The P values were obtained by the log-rank test for comparison of OS.

D-F. Kaplan Meier curve for overall survival (OS) of patients according to *NPM1* positive or negative patients from GSE10358, TCGA-LAML and BEAT-AML datasets. The P values were obtained by the log-rank test for comparison of OS.

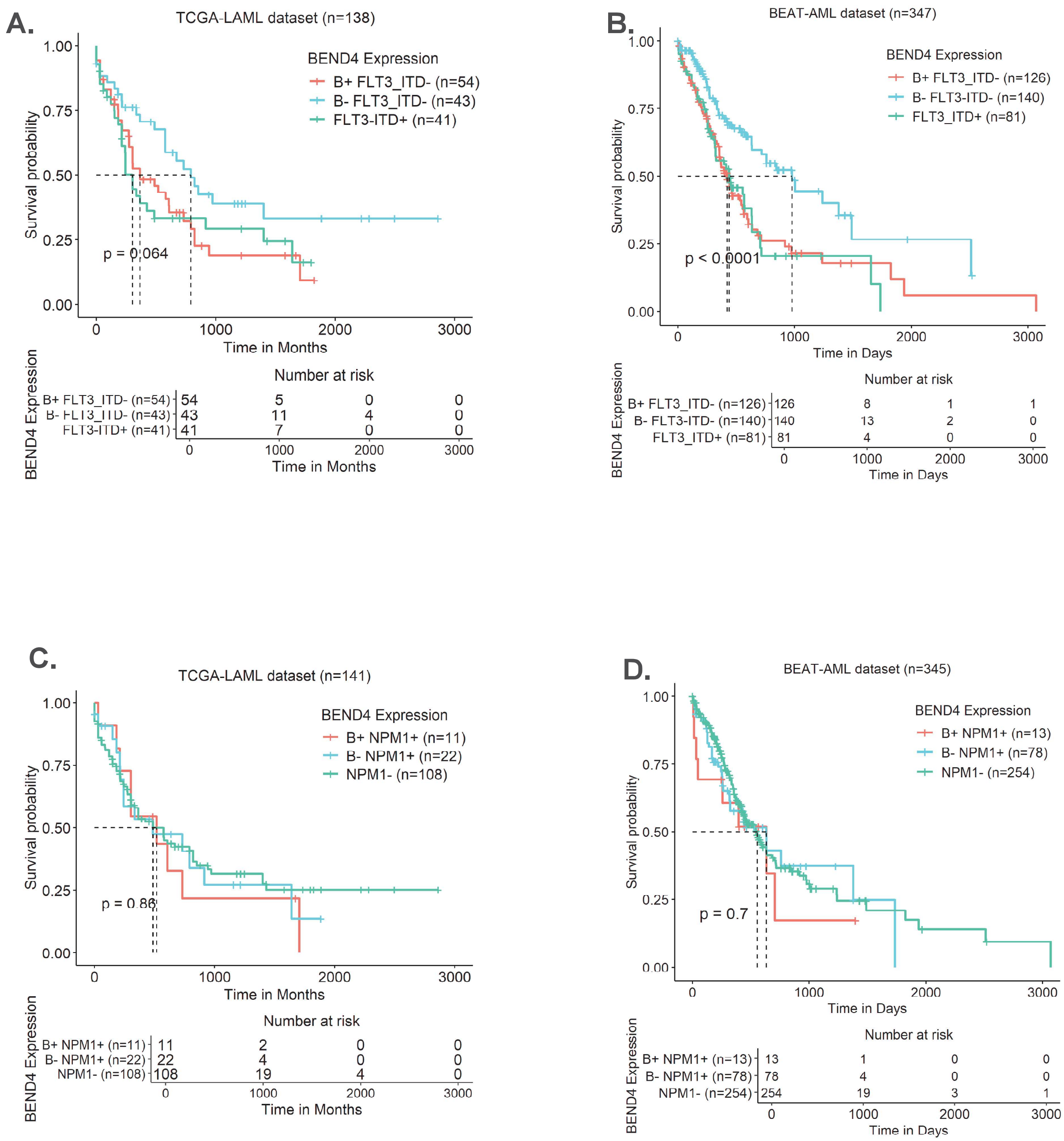

Figure S4: High *BEND4* Expression stratifies *FLT3*-ITD positive and *NPM1*-mutated AML patients

A-B. Kaplan Meier curve for overall survival (OS) of patients according to *FLT3*-ITD positive patients or *BEND4* high and low expression in *FLT3*-ITD negative patients from TCGA-LAML and BEAT-AML datasets. The P values were obtained by the log-rank test for comparison of OS.

C-D. Kaplan Meier curve for overall survival (OS) of patients according to *NPM1* negative patients or *BEND4* high and low expression in *NPM1* positive patients from TCGA-LAML and BEAT-AML datasets. The P values were obtained by the log-rank test for comparison of OS.

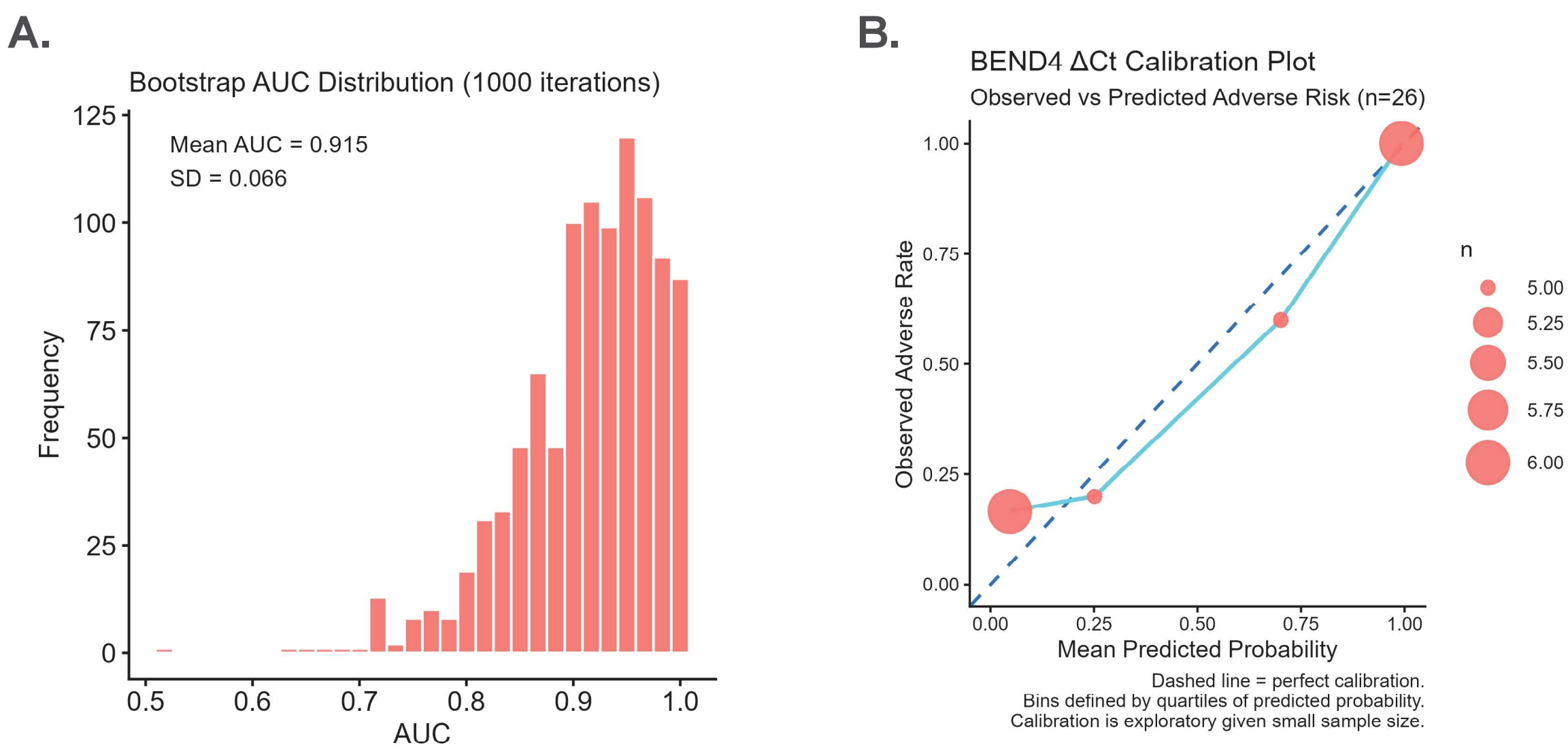

Table S1. Diagnostic performance of BEND4 ΔCt at optimal cutoff (ΔCt < 12.75) in pilot AML cohort (n=26).

Metric	Value	95% CI
Sensitivity	90.90%	-
Specificity	81.80%	-
LR+	5	1.41–17.76
LR-	0.11	0.02–0.74
AUC	0.915*	0.75–1.00*
	*Bootstrap validated (1000 iterations, n=26)	

Figure S5. Validation Analyses for *BEND4* ΔCt Prognostic Assay
A. Bootstrap distribution of AUC values (1000 iterations, n=26) for *BEND4* ΔCt-based risk classification. Mean AUC = 0.915 (SD = 0.066; 95% CI_{percentile}: 0.75–1.00), confirming robust discrimination beyond the point estimate.
B. Calibration plot of predicted vs. observed adverse risk probabilities using logistic regression on *BEND4* ΔCt values (n=26). Points represent risk strata (n=4–10 per bin); close alignment with diagonal demonstrates excellent calibration.

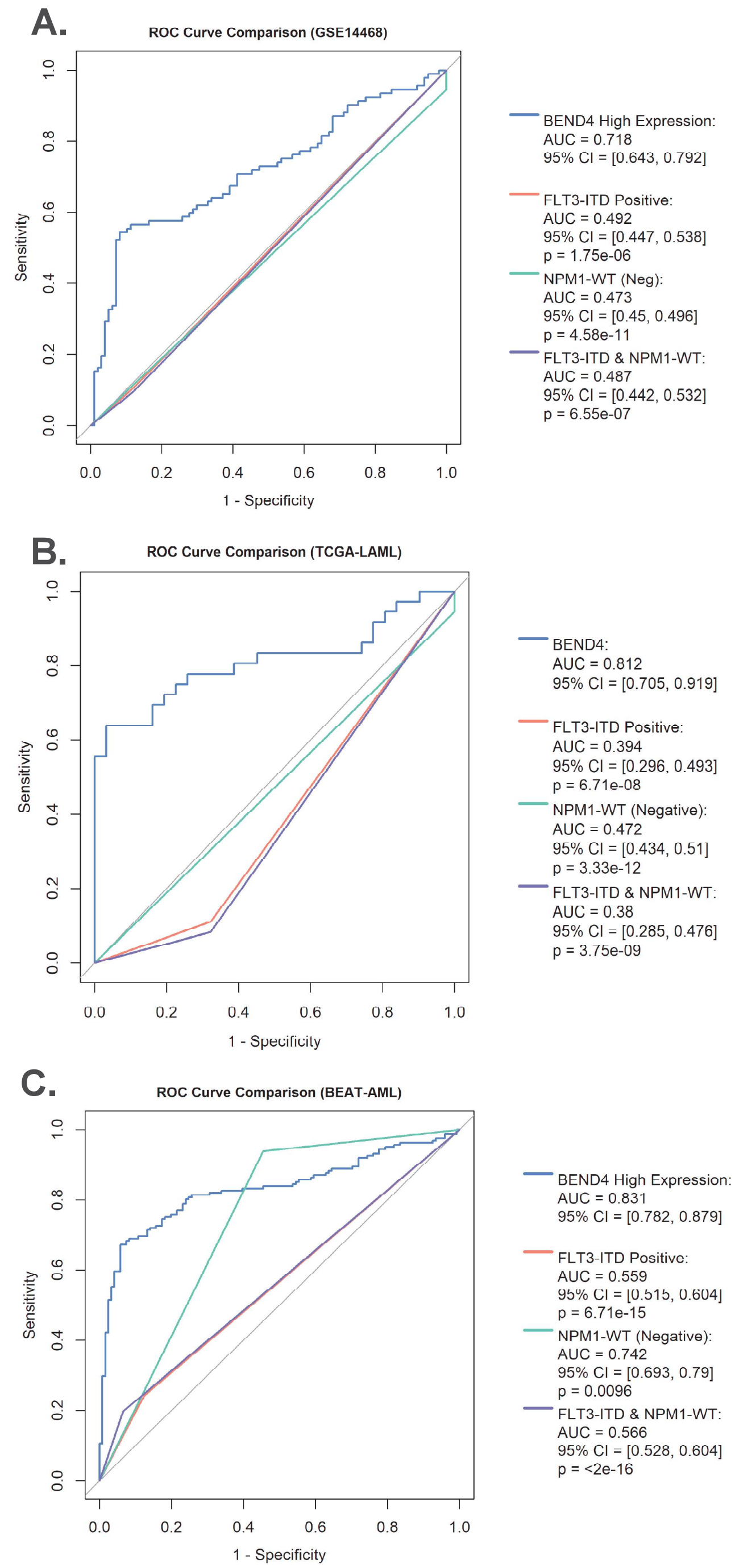

Figure S6: ROC analysis comparing *BEND4* expression with FLT3-ITD and NPM1 mutation status for predicting cytogenetic risk.

A. In GSE14468 dataset *BEND4* showed superior performance (AUC = 0.718) compared to *FLT3*-ITD (AUC = 0.492), *NPM1*-WT (AUC = 0.473), and combined *FLT3*-ITD & *NPM1*-WT (AUC = 0.442), with all comparisons statistically significant (p ≤ 0.05) calculated using DeLong's test for two correlated ROC curves.

B. In TCGA-LAML dataset *BEND4* showed superior performance (AUC = 0.812) compared to *FLT3*-ITD (AUC = 0.394), *NPM1*-WT (AUC = 0.472), and combined *FLT3*-ITD & *NPM1*-WT (AUC = 0.38), with all comparisons statistically significant (p ≤ 0.05) calculated using DeLong's test for two correlated ROC curves.

C. In BEAT-AML dataset *BEND4* showed superior performance (AUC = 0.831) compared to *FLT3*-ITD (AUC = 0.559), *NPM1*-WT (AUC = 0.742), and combined *FLT3*-ITD & *NPM1*-WT (AUC = 0.566), with all comparisons statistically significant (p ≤ 0.05) calculated using DeLong's test for two correlated ROC curves.

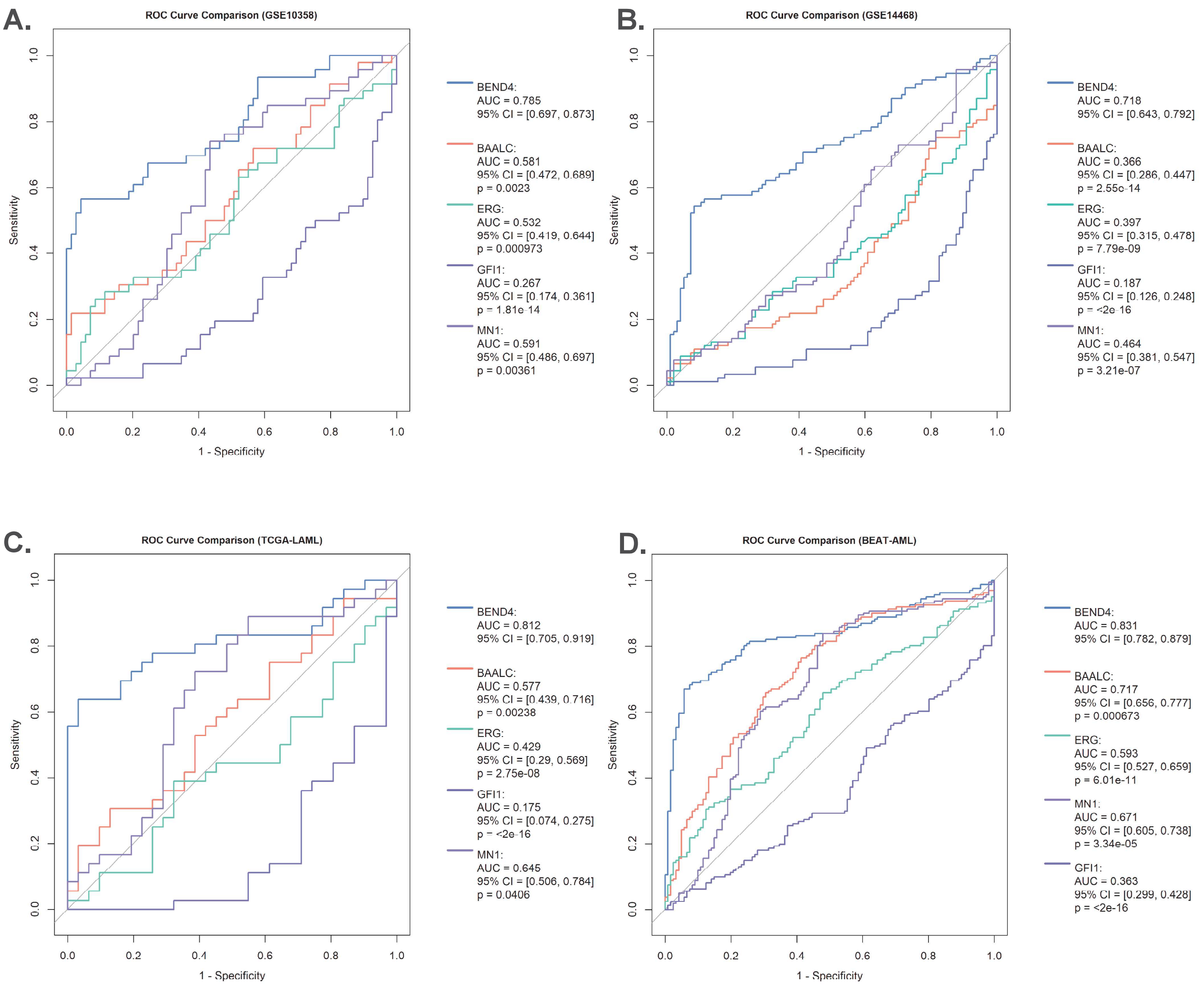

Figure S7: ROC curve analysis comparing the prognostic performance of *BEND4* expression with previously reported gene-based markers—*BAALC*, *ERG*, *MN1*, and *GFI1*—across AML patient datasets. *BEND4* showed significantly higher AUC than all other genes. Statistical significance was assessed using DeLong's test for two correlated ROC curves ($p \leq 0.05$).
A. In GSE10358 dataset, **B.** In GSE14468 dataset, **C.** In TCGA-LAML dataset, **D.** In BEAT-AML dataset

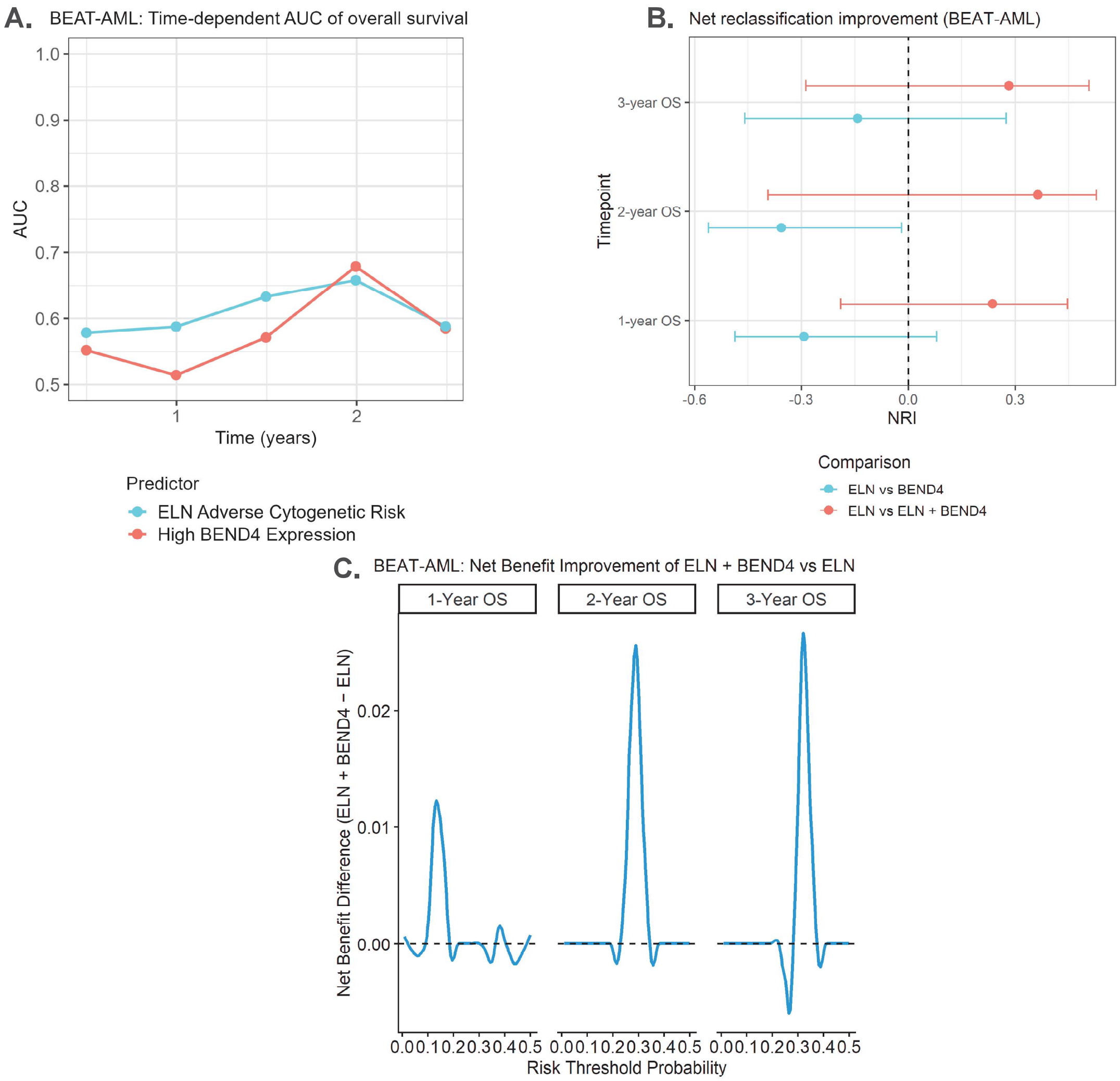

Figure S8. Evaluation of the clinical utility of *BEND4* expression compared with ELN2017 risk classification in the BEAT-AML cohort.

A. Time-dependent ROC analysis showing the predictive performance of *BEND4* expression and ELN adverse cytogenetic risk for overall survival at multiple time points. *BEND4* demonstrates comparable predictive performance to ELN risk classification and shows slightly higher discrimination around the 2-year time point.

B. Net reclassification improvement (NRI) comparing ELN alone with *BEND4* alone and with the combined ELN + BEND4 model at 1-, 2-, and 3-year overall survival. Points represent NRI estimates and horizontal bars indicate 95% confidence intervals. Positive NRI values indicate improved risk classification.

C. Decision curve analysis (DCA) showing the difference in net clinical benefit between the ELN + BEND4 model and ELN alone across a range of risk threshold probabilities for predicting 1-, 2-, and 3-year overall survival. Positive values indicate greater clinical net benefit when *BEND4* expression is incorporated into the ELN model.

Table S2: IC50 values with 95% CI of Doxorubicin and cytarabine in the AML cell lines used in the study

Cell Line	Condition	Doxorubicin IC50 (nM) [95% CI]	Cytarabine IC50 (μM) [95% CI]
MV4-11	Vector Control	47.4 [27.8-76.5]	0.241 [0.186-0.312]
MV4-11	BEND4-OE	797 [447-1375]	2.45 [1.50-4.01]
MOLM13	Vector Control	24.7 [18.1-30.7]	0.108 [0.097-0.118]
MOLM13	BEND4-OE	399 [330-484]	2.79 [2.02-4.13]
THP-1	Scrambled	241 [207-282]	1.12 [0.782-1.61]
THP-1	BEND4-KD	37.9 [22.9-60.8]	0.273 [0.193-0.387]

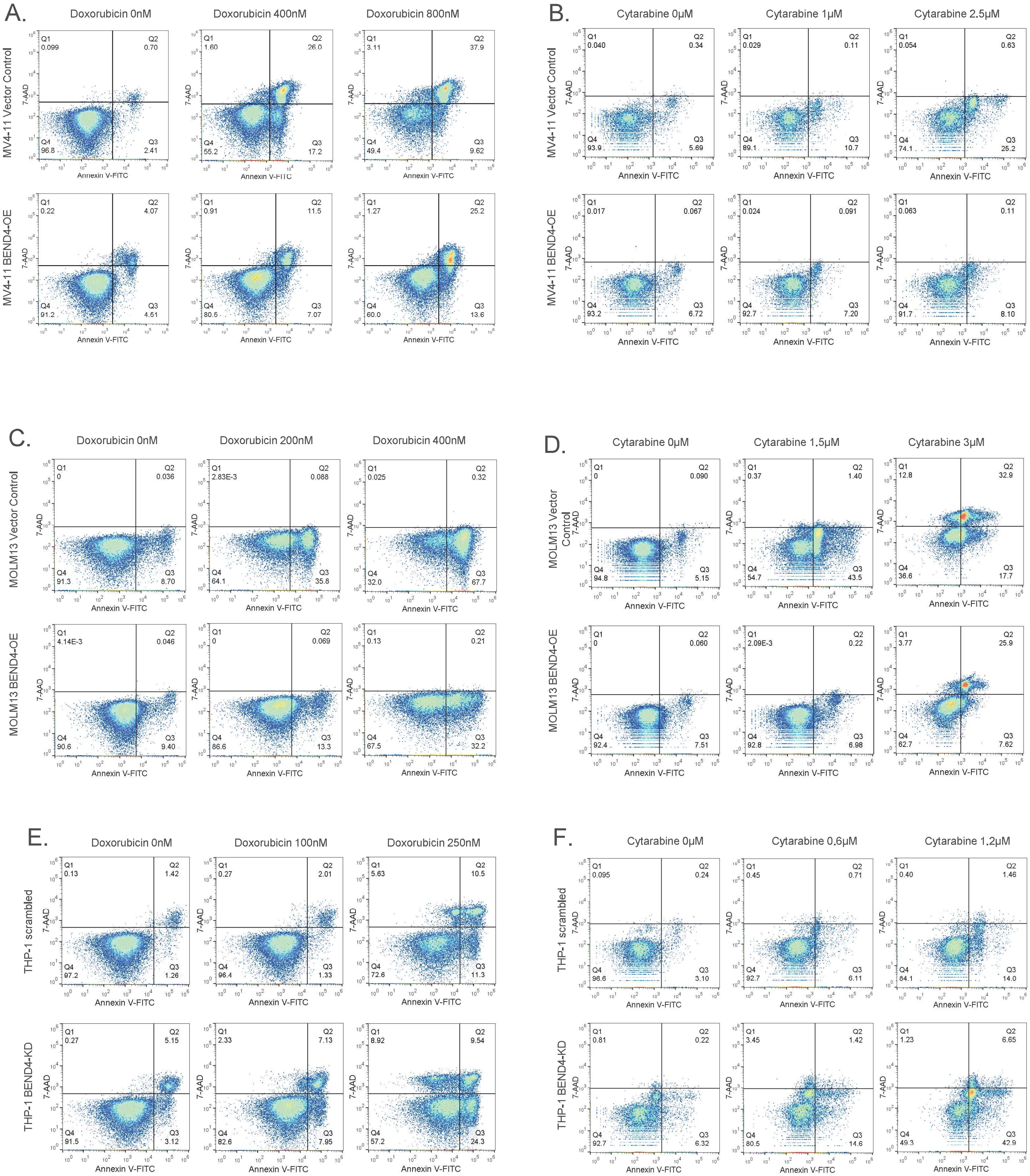

Figure S9. Representative flow cytometry dot plots for Annexin V/7-AAD apoptosis assay in BEND4-perturbed AML cell lines following chemotherapy treatment.

Representative dot plots from one biological replicate are shown for each condition. Annexin V-FITC is on the x-axis and 7-AAD on the y-axis. The lower-right and upper-right quadrants represent early apoptotic (Annexin V+/7-AAD−) and late apoptotic/necrotic (Annexin V+/7-AAD+) cells, respectively. The percentage of total apoptotic cells (early + late) from three independent biological replicates is quantified in Figure 4A, B, E, F, I, and J.

A–B. MV4-11 vector control and BEND4-OE cells treated with doxorubicin (0, 400, and 800 nM) and cytarabine (Ara-C; 0, 1, and 2.5 μM), respectively.

C–D. MOLM13 vector control and BEND4-OE cells treated with doxorubicin (0, 200, and 400 nM) and cytarabine (Ara-C; 0, 1.5, and 3 μM), respectively.

E–F. THP-1 scrambled control and BEND4-KD cells treated with doxorubicin (0, 100, and 250 nM) and cytarabine (Ara-C; 0, 0.6, and 1.2 μM), respectively.

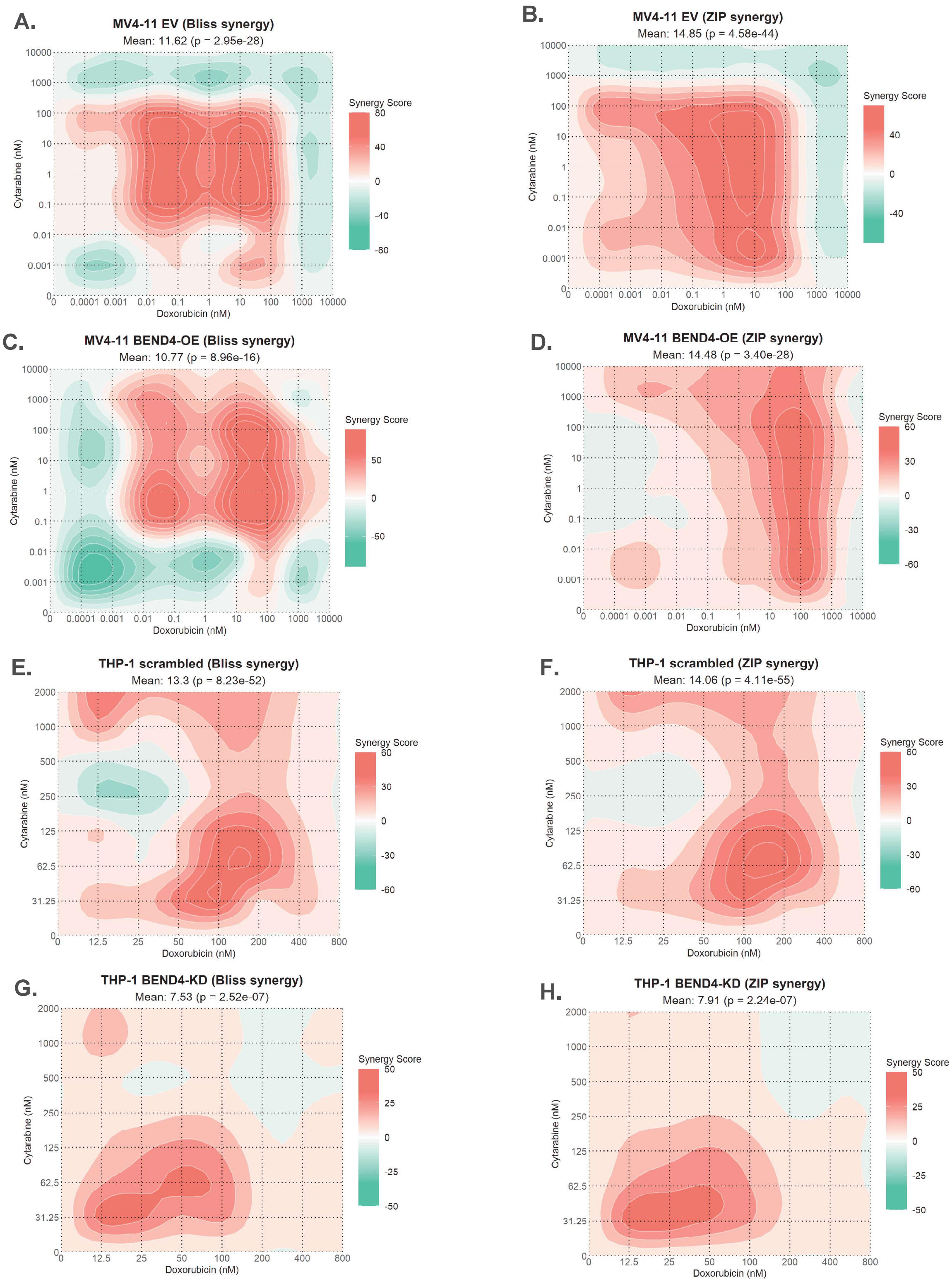

Figure S10. Synergy analysis of doxorubicin and cytarabine combination treatment in AML cells. Bliss and ZIP synergy heatmaps showing the combinatorial effects of doxorubicin and cytarabine (Ara-C) after 48 h treatment in MV4-11 vector control (EV) and BEND4-overexpressing cells **(A–D)**, and THP-1 scrambled control and BEND4 knockdown cells **(E–H)**. Mean synergy scores are indicated within each panel, with positive values and significant p-values representing synergistic interactions. The Bliss/ZIP synergy and p-values were calculated from the MTT assay data using synergyfinder R package.

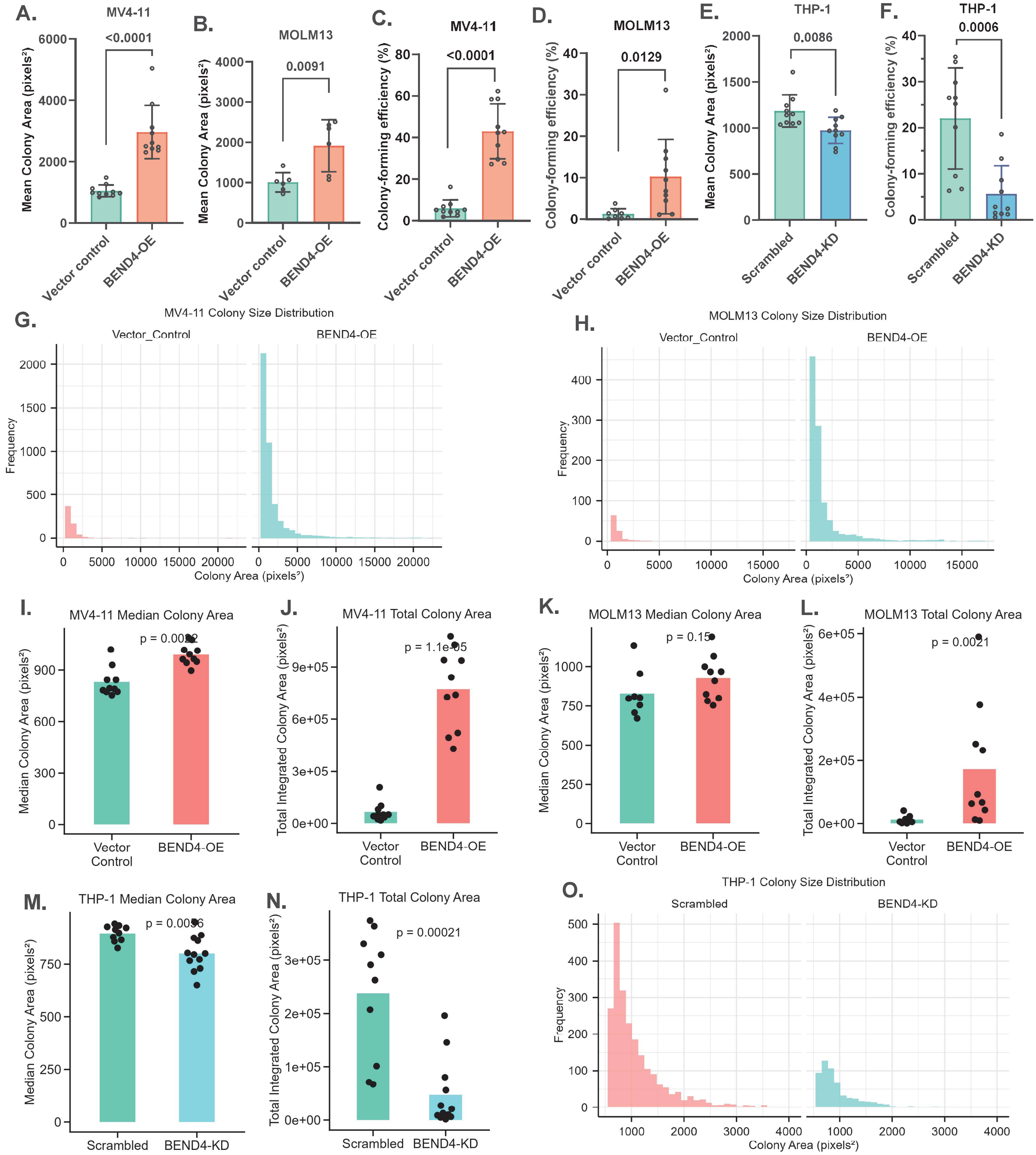

Figure S11. Quantitative analysis of clonogenic capacity in BEND4-overexpressing and BEND4-knockdown AML cells.

(A–B) Mean colony area (pixels²) in MV4-11 **(A)** and MOLM13 **(B)** cells overexpressing BEND4 (BEND4-OE) compared to empty vector control (Vector Control). **(C–D)** Colony-forming efficiency (%) in MV4-11 **(C)** and MOLM13 **(D)** BEND4-OE and Vector Control cells. **(E)** Mean colony area and **(F)** colony-forming efficiency (%) in THP-1 cells with BEND4 knockdown (BEND4-KD) compared to scrambled control. Bar plots represent mean ± SD of biological replicates; individual data points represent per-well values. Statistical significance was determined by unpaired two-tailed Student's t-test; p-values are indicated on the plots**. (G–H)** Colony size distribution histograms showing the frequency of individual colony areas (pixels²) in MV4-11 **(G)** and MOLM13 **(H)** BEND4-OE and Vector Control cells**. (I–J)** Per-well median colony area **(I)** and total integrated colony area **(J)** in MV4-11 BEND4-OE versus Vector Control cells. **(K–L)** Per-well median colony area **(K)** and total integrated colony area **(L)** in MOLM13 BEND4-OE versus Vector Control cells. **(M-N)** Per-well median colony area **(M)** and total integrated colony area **(N)** in THP-1 BEND4-KD versus scrambled control cells. **(O)** Colony size distribution histogram in THP-1 BEND4-KD and scrambled control cells. Bar plots in I–L and N–O represent mean of per-well values; individual data points represent individual wells. Statistical significance for median and total colony area comparisons was determined by Wilcoxon rank-sum test; p-values are indicated on the plots. Top 1% of colony areas were removed as outliers prior to analysis.

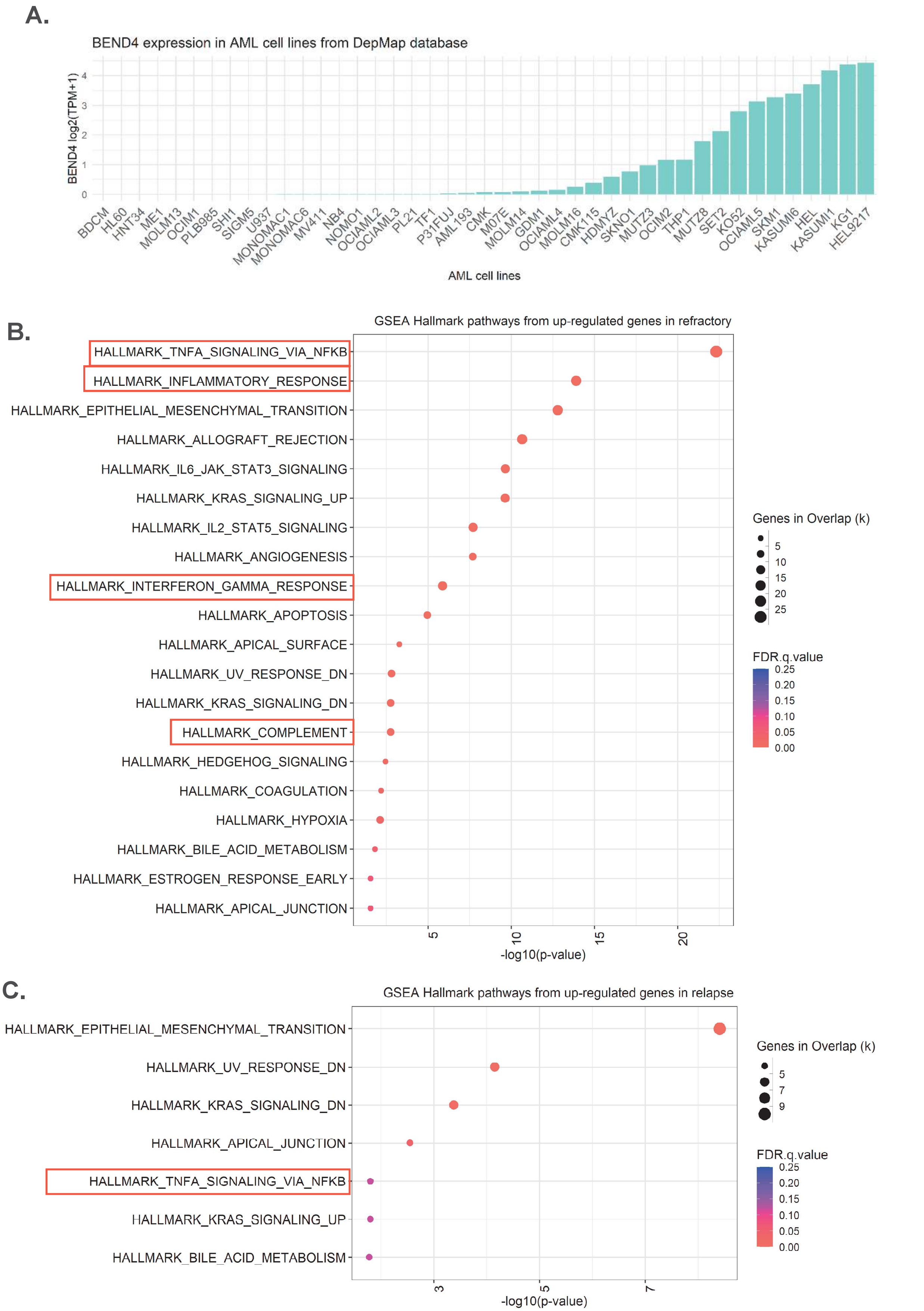

Figure S12: *BEND4* expression and pathway enrichment of up-regulated genes in the relapse and refractory AML patients

A. *BEND4* mRNA expression in different AML cell lines from DepMap database.

B-C. Dot plot showing hallmark pathway enrichment for upregulated genes in the refractory and relapse patients from BEAT-AML and GSE165430 dataset respectively.

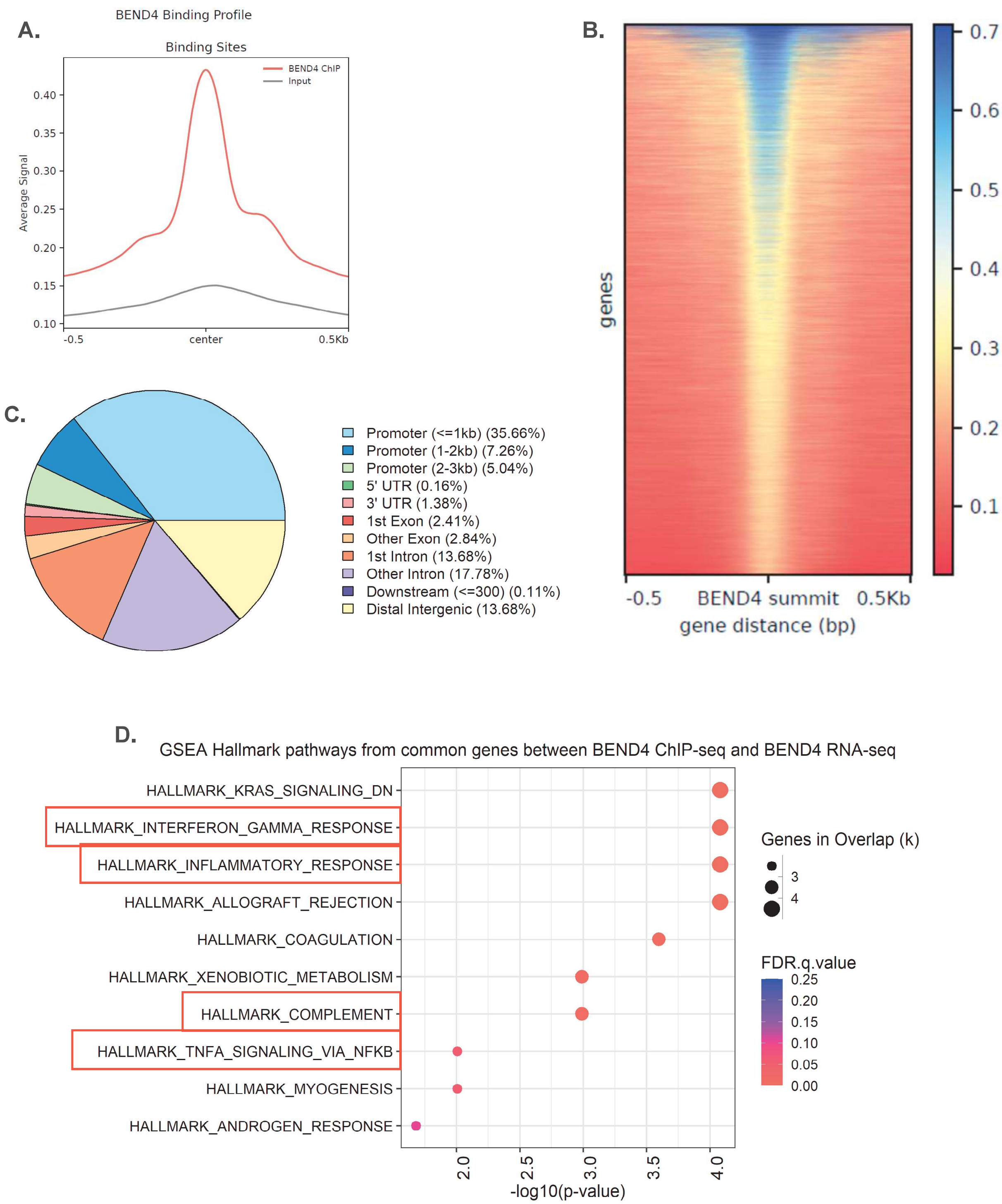

Figure S13. BEND4 ChIP-seq peak profiling and genomic distribution in MV4-11 *BEND4*-OE cells and gene set enrichment analysis

A. Metaplots showing BEND4 ChIP-seq signal enrichment (red) versus input control (gray) centered on peak summits, revealing focused binding.
B. Heatmap of normalized BEND4 signal intensity across all called peaks, demonstrating strong central enrichment relative to gene distance.
C. Genomic distribution of BEND4 peaks, with promoter-proximal regions (≤1 kb of TSS: 35.7%; 1–2 kb: 7.3%; 2–3 kb: 5.0%) predominating, followed by introns (1st intron: 13.7%; other introns: 17.8%), distal intergenic (13.7%), exons (1st exon: 2.4%; other exons: 2.8%), UTRs (5′ UTR: 0.2%; 3′ UTR: 1.4%), and downstream (<300 bp: 0.1%).
D. Dot plot showing hallmark pathway enrichment for 96 common genes between RNA-seq and ChIP-seq.

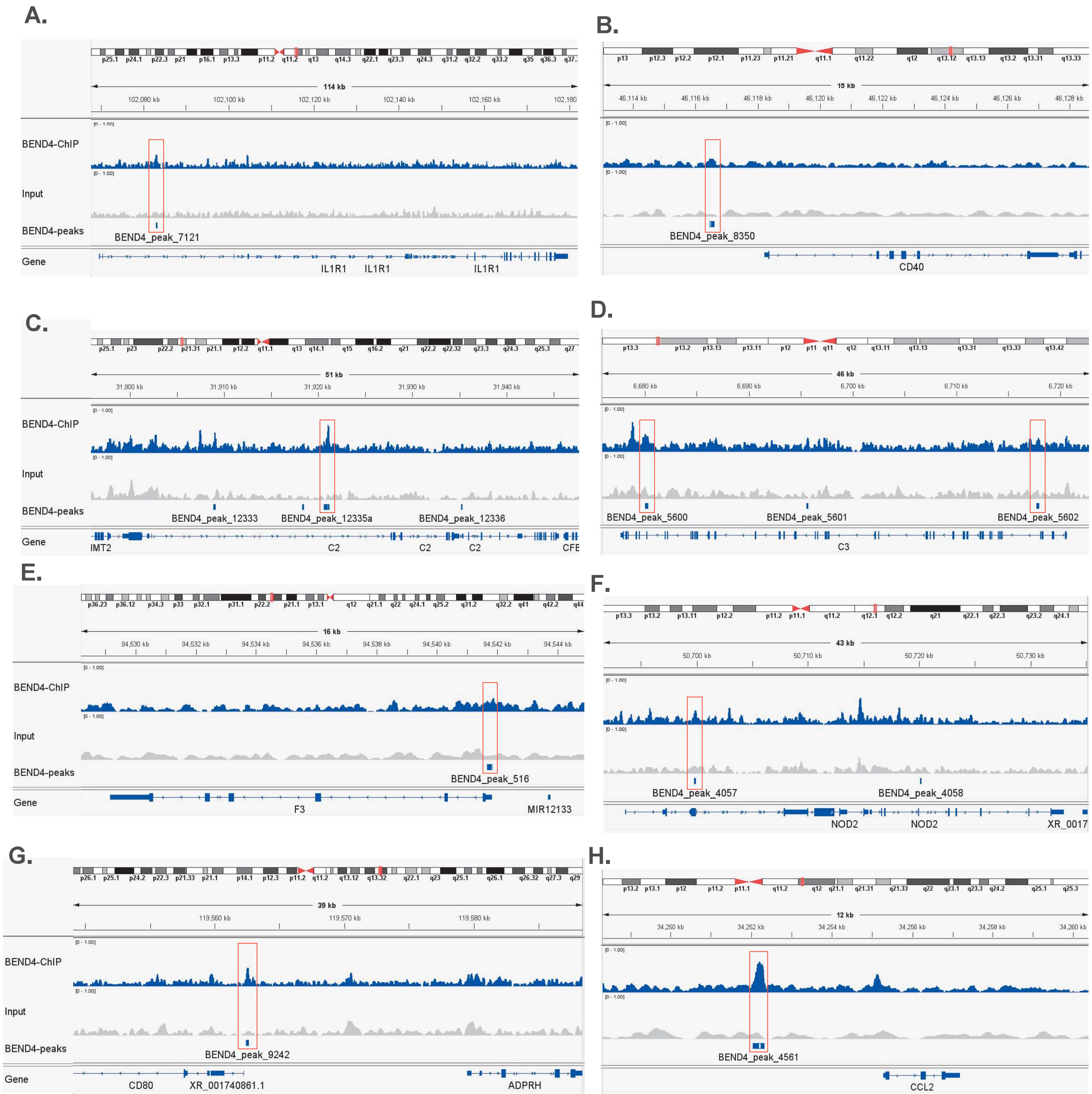

Figure S14. BEND4 enrichment at promoters of immune and inflammatory signaling genes
A-H. Genome browser views showing BEND4 ChIP-seq signal (blue) and input control (grey) across the indicated genomic loci. The highlighted regions denote significant BEND4 binding peaks identified by MACS2 within promoter or gene body regions of the *IL1R1*, *CD40*, *C2*, *C3*, *F3*, *NOD2*, *CD80*, and *CCL2*

Table S3: BEND4 occupancy in the enriched pathway genes

Gene Name	Fold enrichment	p-value	q-value	Binding site	Pathways
IL1R1	4.4608	0.0000	0.0000	Gene body	Inflammatory response pathway, TNF-α signaling via NF-κB
CD40	3.30293	0.0000	0.0166	Promoter	IFN-γ response pathway, Inflammatory response pathway
C2	6.11399	0.0000	0.0000	Promoter	Complement pathway
C3	3.74784	0.0000	0.0005	Promoter, gene body	Complement pathway
F3	2.91428	0.0001	0.0253	Promoter	Complement pathway, Inflammatory response pathway, TNF-α signaling via NF-κB
NOD2	3.14033	0.0000	0.0047	Promoter	Inflammatory response pathway
CD80	5.67921	0.0000	0.0000	Promoter	TNF-α signaling via NF-κB
CCL2	5.97882	0.0000	0.0000	Promoter	IFN-γ response pathway, Inflammatory response pathway, TNF-α signaling via NF-κB

Table S4. List of Oligonucleotides (5' to 3')

qPCR primers	
Primer Name	Sequence (5'-3')
BEND4_Forward	GGAAAAGGAAAAGGTCAGTGC
BEND4_Reverse	GTTTATCTGCTCTTCCGAGGG
GAPDH_Forward	GCACCGTCAAGGCTGAGAAC
GAPDH_Reverse	AGGGATCTCGCTCCTGGAA
RPL19_Forward	CAAGCGGATTCTCATGGAACA
RPL19_Reverse	TGGTCAGCCAGGAGCTTCTT
IL1R1_Forward	GTGCTTTGGTACAGGGATTCCTG
IL1R1_Reverse	CACAGTCAGAGGTAGACCCTTC
CD40_Forward	CCTGTTTGCCATCCTCTTGGTG
CD40_Reverse	AGCAGTGTTGGAGCCAGGAAGA
C2_Forward	GGTCCAAGAAGGATGGTGAGAG
C2_Reverse	GCAGATGGTGTCTGTGAGCTTG
MAP4K1_Forward	CAAGATCCAGGACACCAAAGGC
MAP4K1_Reverse	ACCACTGAAGCAGGACAACGGA
F3_Forward	CAGAGTTCACACCTTACCTGGAG
F3_Reverse	GTTGTTCCTTCTGACTAAAGTCCG
C3_Forward	GTGGAAATCCGAGCCGTTCTCT
C3_Reverse	GATGGTTACGGTCTGCTGGTGA
NOD2_Forward	GCACTGATGCTGGCAAAGAACG
NOD2_Reverse	CTTCAGTCCTTCTGCGAGAGAAC

Supplementary Data Legend

Supplementary Data 1 : Bivariate association analysis of *BEND4* expression with AML patients' clinical characteristics in four AML datasets (TCGA-LAML, BeatAML, GSE10358, and GSE14468).

Unprocessed western blots

Fig 3G.

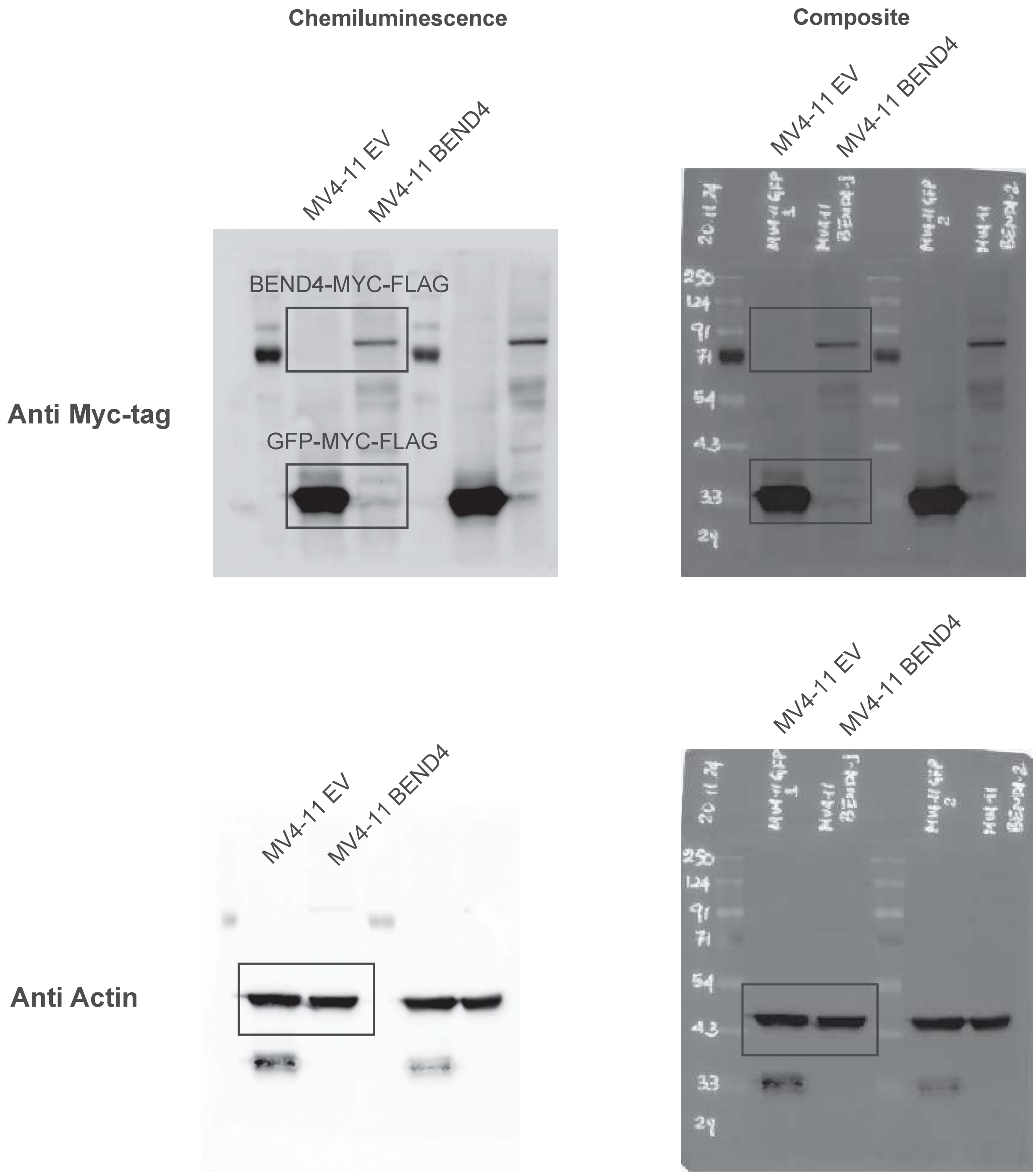

Unprocessed western blots

Fig 3H.

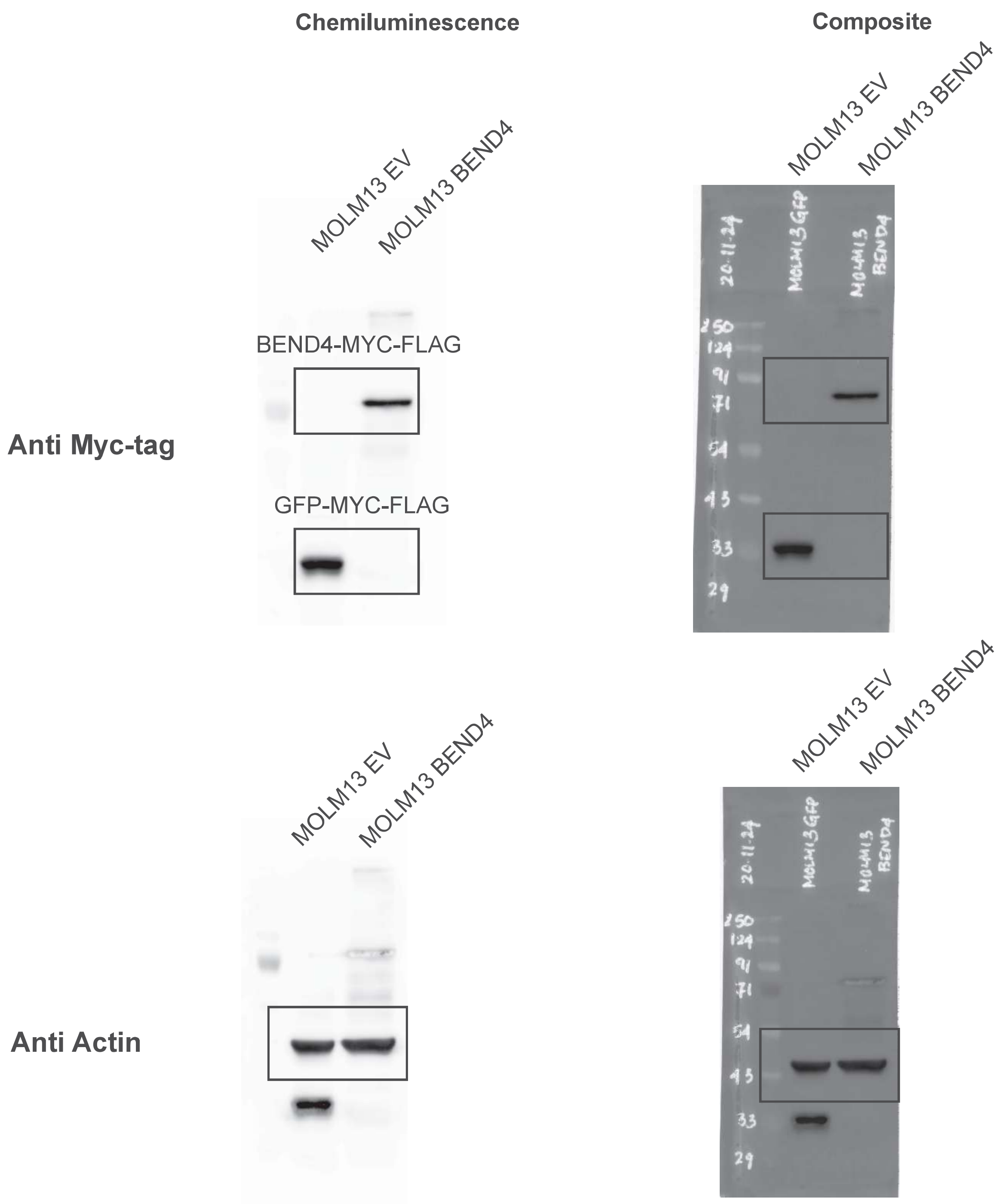

Unprocessed western blots

Fig 5A.

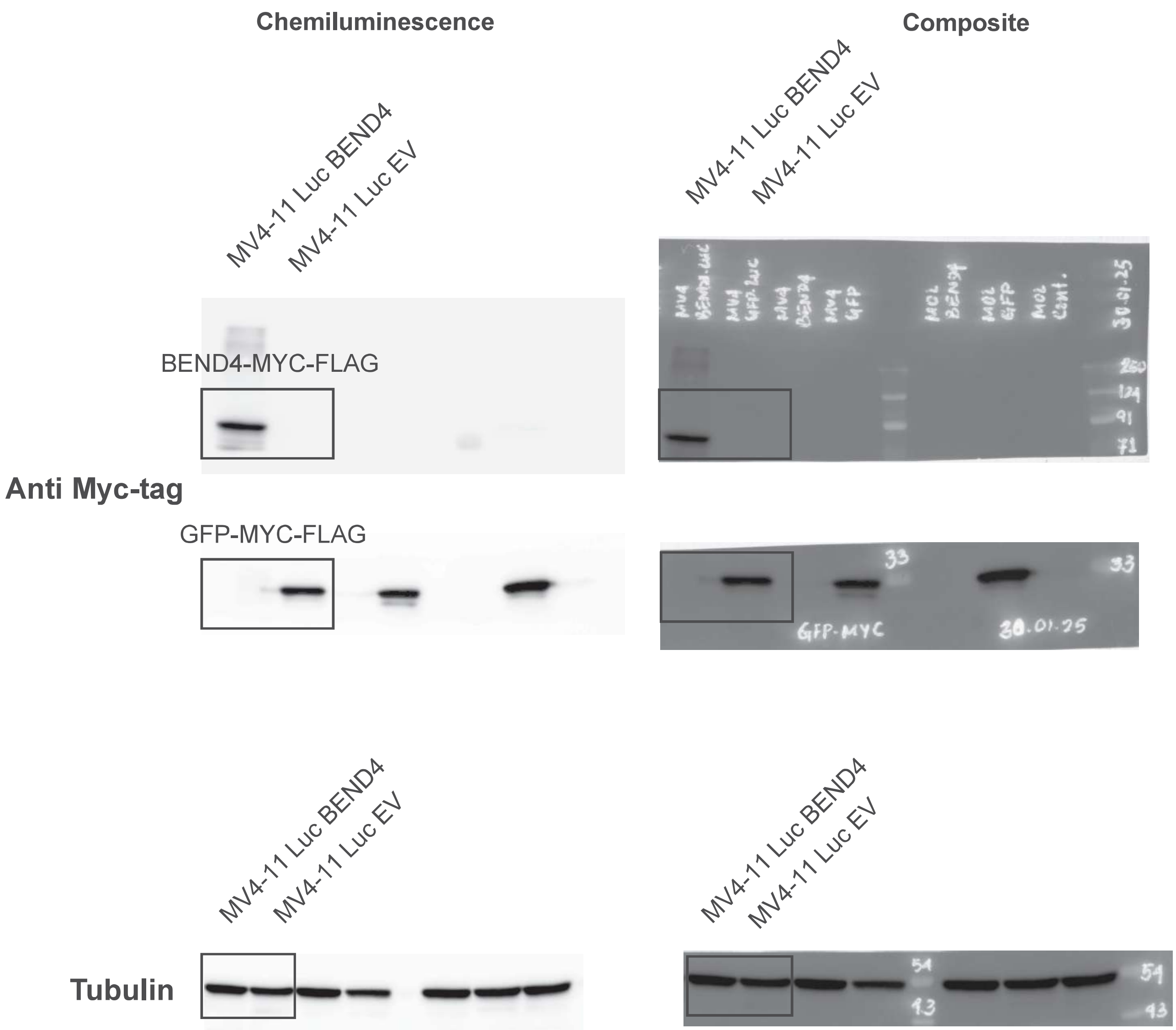

Supplement: Supplementary file 1 [file 41698_2026_1534_MOESM1_ESM.pdf]
